# Attenuated vaccine PmCQ2Δ4555–4580 effectively protects mice against *Pasteurella multocida* infection

**DOI:** 10.1186/s12917-024-03948-6

**Published:** 2024-03-09

**Authors:** Fang He, Pan Xiong, Huihui Zhang, Liu Yang, Yangyang Qiu, Pan Li, Guangfu Zhao, Nengzhang Li, Yuanyi Peng

**Affiliations:** 1 College of Veterinary Medicine, Southwest University, Chongqing, 400715 China; 2 Department of Environment and Safety Engineering, Taiyuan institute of technology, Taiyuan, 030008 China

**Keywords:** *Pasteurella multocida*, Infection, Vaccine, Immune protection

## Abstract

*Pasteurella multocida* type A (PmA) mainly causes respiratory diseases such as pneumonia in bovines, leading to great economic losses to the breeding industry. At present, there is still no effective commercial vaccine against PmA infection. In this study, a mutant strain (PmCQ2Δ4555–4580) with brand-new phenotypes was obtained after serially passaging at 42 °C. Whole genome resequencing and PCR analysis showed that PmCQ2Δ4555–4580 missed six genes, including PmCQ2_004555, PmCQ2_004560, PmCQ2_004565, PmCQ2_004570, PmCQ2_004575, and PmCQ2_004580. Importantly, the virulence of PmCQ2Δ4555–4580 was reduced by approximately 2.8 × 10^9^ times in mice. Notably, live PmCQ2Δ4555–4580 could provide 100%, 100% and 40% protection against PmA, PmB and PmF, respectively; and inactivated PmCQ2Δ4555–4580 could provide 100% and 87.5% protection against PmA and PmB. Interestingly, immune protection-related proteins were significantly upregulated in PmCQ2Δ4555–4580 based on RNA-seq and bioinformatics analysis. Meaningfully, by in vitro expression, purification and in vivo immunization, 12 proteins had different degrees of immune protective effects. Among them, PmCQ2_008205, PmCQ2_010435, PmCQ2_008190, and PmCQ2_004170 had the best protective effect, the protection rates against PmA were 50%, 40%, 30%, and 30%, respectively, and the protective rates against PmB were 62.5%, 42.9%, 37.5%, and 28.6%, respectively. Collectively, PmCQ2Δ4555–4580 is a potential vaccine candidate for the prevention of Pasteurellosis involving in high expression of immune protective related proteins.

**Supplementary Information:**

The online version contains supplementary material available at 10.1186/s12917-024-03948-6.

## Background

*Pasteurella multocida* (Pm), a gram-negative pathogenic bacterium, is divided into A, B, D, E, and F 5 serotypes according to the difference in capsule [1]. Among them, Pm serotype A (PmA) mainly causes respiratory syndrome [2], Pm serotype B (PmB) usually results in hemorrhagic septicemia [3], and Pm serotype F (PmF) most commonly leads to avian cholera [4], but PmF has been recently isolated from the lungs of pneumonic rabbits [5] and pigs [6]. Frighteningly, Pm can lead to a variety of diseases in many animals (e.g., poultry, livestock, wild animals, and even humans) [7], causing enormous economic losses and serious public health problems. At present, the prevention and control of Pm mainly rely on vaccines and antibiotics. However, antibiotics have problems such as high price, drug resistance and environmental pollution [8, 9]. Thus, prophylactic immunization is a safe and effective preventive measure in the case of Pasteurellosis.

Vaccination with live attenuated vaccines, inactivated vaccines, and subunit vaccines is an effective and economical way to protect animals from Pm infection [10]. However, the development of bovine-derived Pm vaccines is still in its infancy [11]. Currently, the existing commercialized Pm vaccines are specific for PmB, and less for PmA. Additionally, there is no cross-protective vaccine against multiple different serotypes of Pm in clinical, which brings certain challenges to the prevention and control of the disease [12]. In recent years, there has been widespread use of genetic modification technology to obtain mutation-attenuated live vaccines, which provides a reference for determining new vaccine candidates [13, 14]. However, there are no new vaccines with good cross-immune protection produced under new strategies [12]. In addition to virulence gene deletion, the use of chemically mutagenic substances, physical methods and continuous biological subgenerations are all strategies for attenuated vaccine preparation [12]. For example, BCG, a vaccine for the prevention of tuberculosis, was obtained by serially passaging on potato slices soaked in ox bile and glycerol [15]. Overall, the goal of the study is to develop an attenuated vaccine that can protect against the majority of infections due to Pm.

In this study, we occasionally found a mutant strain with a different colony morphology when compared to PmCQ2, and identified 6 gene deletions that could contribute to these morphological changes by whole-genome resequencing. Then, the virulence of the mutant strain PmCQ2Δ4555–4580 was compared with that of PmCQ2 in mouse models, which was drastically reduced. Importantly, PmCQ2Δ4555–4580 showed tremendous immunogenicity and protective effects in Pm infection models. Finally, the possible reasons for the increased immune protection of PmCQ2Δ4555–4580 were investigated by transcriptome sequencing, bioinformatics analysis and subunit vaccine immunization. Taken together, the results indicate that PmCQ2Δ4555–4580 is a promising candidate vaccine against Pm infection.

## Results

### The characteristics of the mutant strain PmCQ2Δ4555–4580

**Fig. 1:**
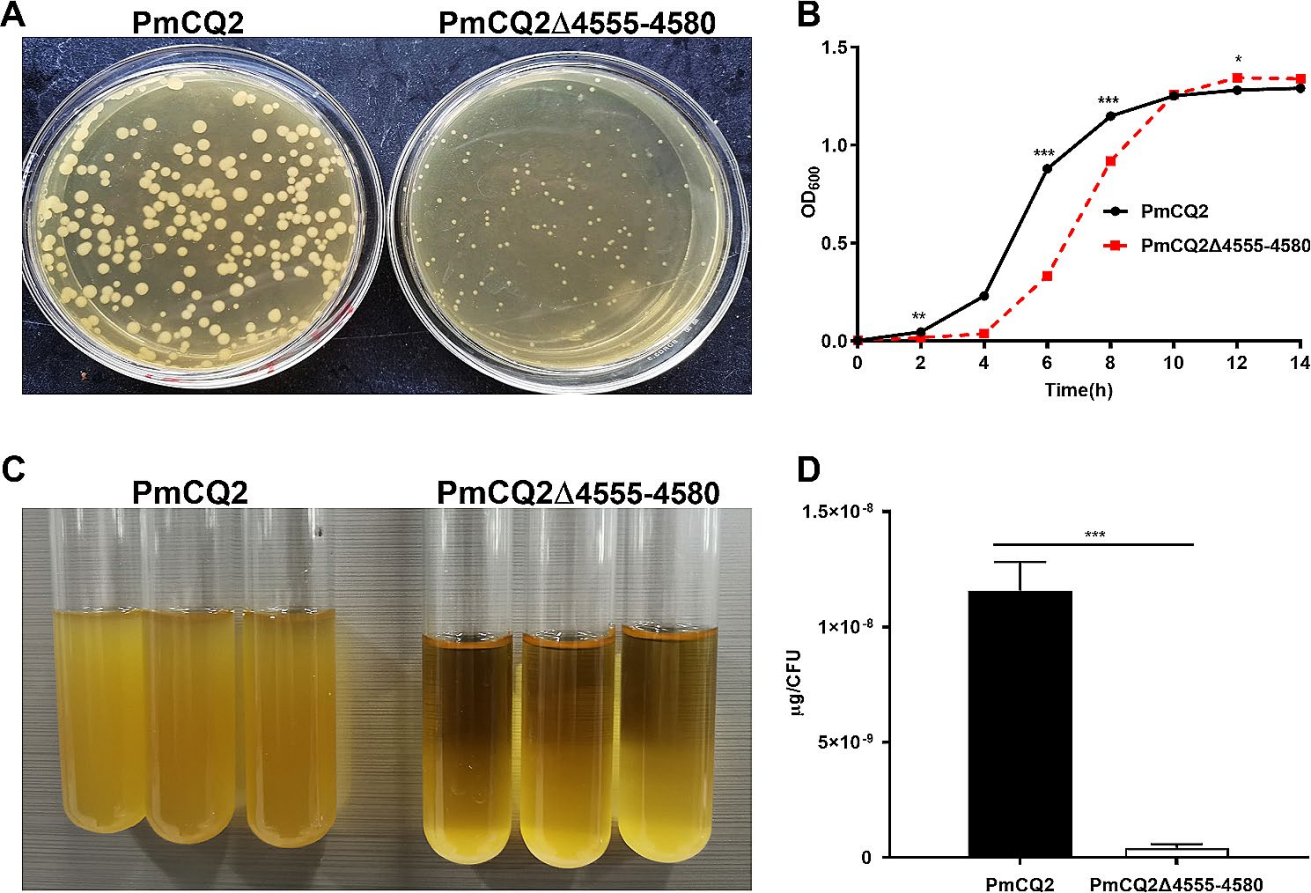
**The characteristics of mutant strain PmCQ2Δ4555–4580.** **A**: Colony morphology of PmCQ2, and PmCQ2Δ4555–4580. **B**: Bacterial growth curves of PmCQ2, and PmCQ2Δ4555–4580 based on OD_600_. **C**: The precipitation condition of PmCQ2, and PmCQ2Δ4555–4580 at 10,000 rpm for 5 min. **D**: The content of capsule polysaccharide in PmCQ2, and PmCQ2Δ4555–4580. The data of B and D were pooled from three independent experiments with 6 replicates per group, and B was analyzed by multiple comparative analysis, and expressed as means ± SD. *P < 0.05, **P < 0.01, ***P < 0.001

After serially passaging in vitro at 42 °C, we occasionally found a PmCQ2-origin mutant strain with a smaller colony morphology (Fig. 1A). We also noticed that the mutation strain showed obviously different growth curves compared with PmCQ2, as evidenced by the inhibition of growth at 0–10 h (Fig. 1B). Additionally, unlike PmCQ2, PmCQ2Δ4555–4580 was easier to centrifuge (Supplementary Fig. 1A) and precipitate (Fig. 1C) to the bottom of the tube, implying that the ability to produce capsules could be impaired in this mutant strain. Accordingly, capsule content was indeed significantly decreased in PmCQ2Δ4555–4580 (Fig. 1D). Moreover, PmCQ2Δ4555–4580 was stable for more than 50 passages (Supplementary Fig. 1). Together, the mutation strain PmCQ2Δ4555–4580 exhibited a brand-new phenotype.

### Identification of the of mutant strain PmCQ2Δ4555–4580

**Fig. 2:**
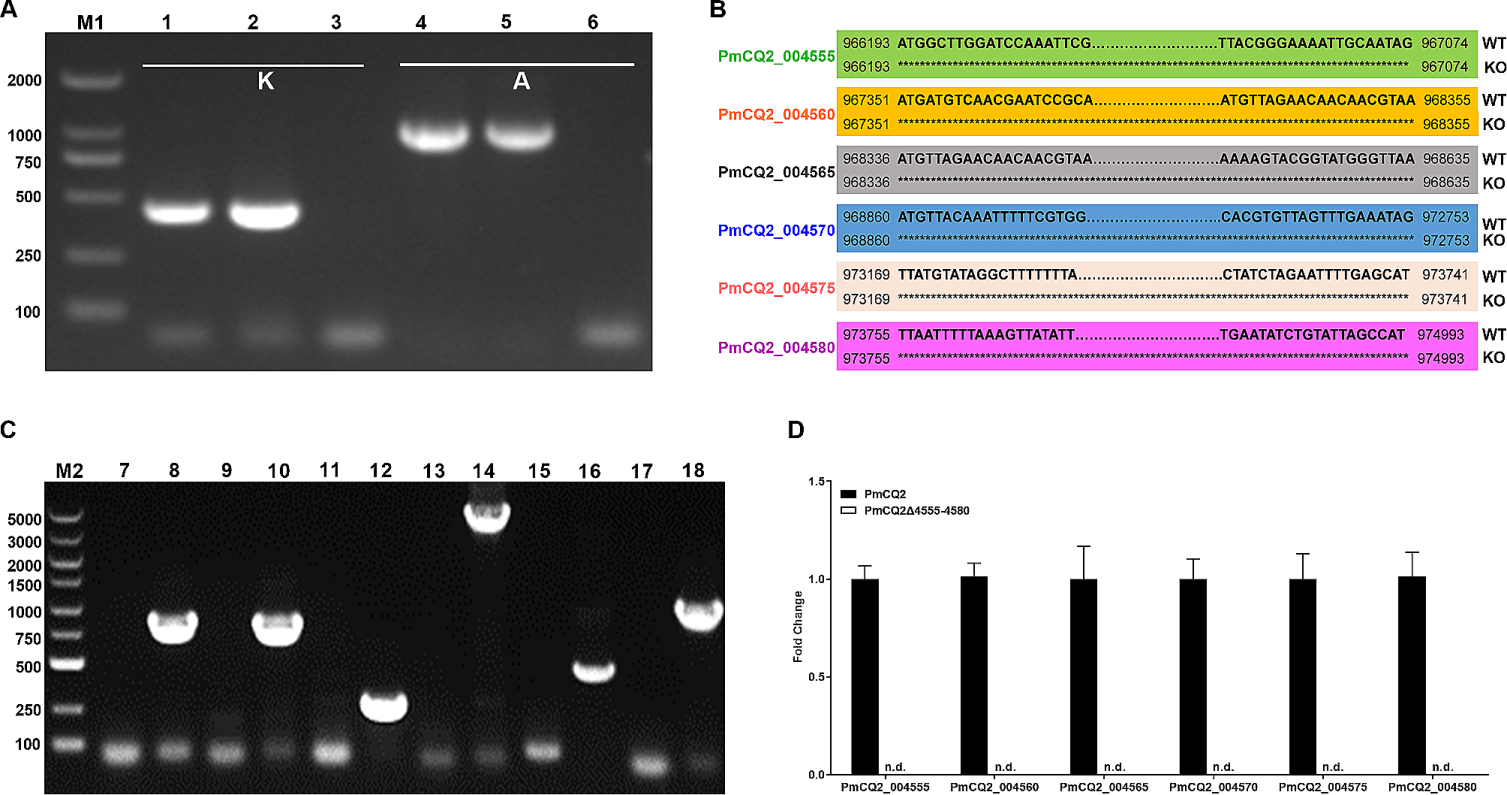
**The identification of PmCQ2Δ4555–4580.** **A**: PCR identification of PmA. **B**: Whole genome resequencing of PmCQ2Δ4555–4580. **C**: PCR confirmation of missed genes in PmCQ2Δ4555–4580. **D**: RT-qPCR confirmation of missed genes in PmCQ2Δ4555–4580. M1: 2000 DNA marker. M2: 5000 DNA marker. Lanes 1, 4, 8, 10, 12, 14, 16, and 18: PmCQ2. Lanes 2, 5, 7, 9, 11, 13, 15, and 17: PmCQ2Δ4555–4580. Lanes 3 and 6: *E. coli*. K: The specific primer (KMT1-F/R) of Pm. A: The specific primer (CapA-F/R) of PmA. The data of D were pooled from three independent experiments with 3 replicates per group, and were analyzed by multiple comparative analysis, and expressed as means ± SD

To rule out other bacterial contamination, specific primer PCR was used to identify and find that PmCQ2Δ4555–4580 belongs to PmA (Fig. 2A). Furthermore, the results of whole-genome resequencing revealed that PmCQ2Δ4555–4580 was the wild-type strain PmCQ2, with six obvious genes missing (Supplementary Table 4), including PmCQ2_004555 (50 S ribosomal protein L11 methyltransferase), PmCQ2_004560 (tRNA dihydrouridine synthase DusB), PmCQ2_004565 (Fis family transcriptional regulator), PmCQ2_004570 (phosphorribosylformyl-glycinamidine synthase), PmCQ2_004575 (hypothetical protein), and PmCQ2_004580 (DUF 26-containing protein) (Fig. 2B). Then, the 6 missing genes were identified by PCR and RT-qPCR, which is consistent with our whole-genome resequencing data (Fig. 2C, D). As a result, the above findings indicated that PmCQ2Δ4555–4580 is a mutant of the wild-type strain PmCQ2.

### The pathogenicity of PmCQ2Δ4555–4580

**Fig. 3:**
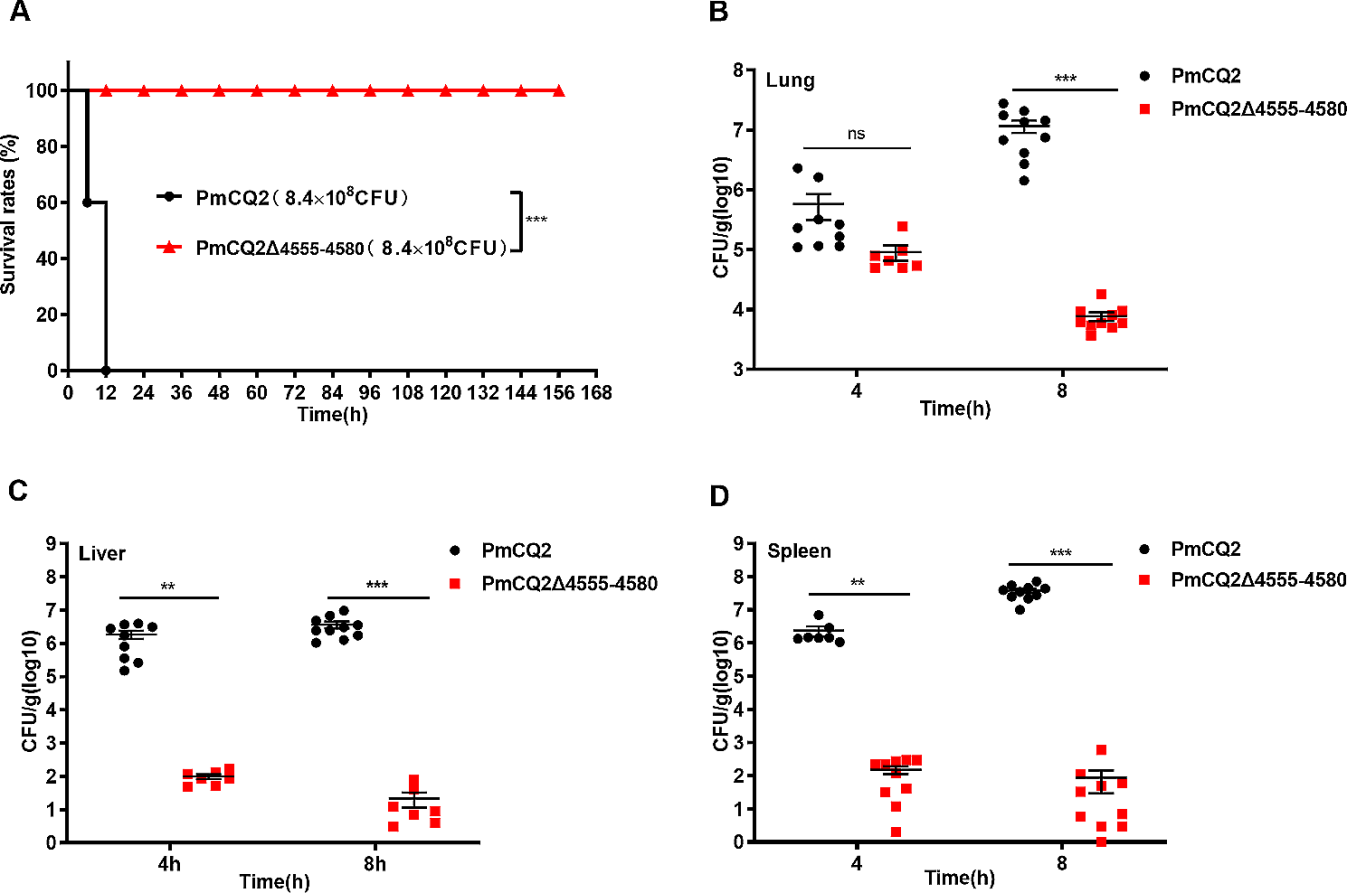
**The virulence of PmCQ2Δ4555–4580.** **A**: Survival rates of mice. **B-D**: Bacterial burdens in the lungs **(B)**, liver **(C)** and spleen **(D)** of mice at 4, and 8 h postinfection. A was pooled from three independent experiments with 10 replicates per group, and determined by Log-rank (Mantel-Cox) test. B-D were pooled from two independent experiments with 7–10 replicates per group, and were analyzed by two-way ANOVA, and expressed as means ± SD. **P < 0.01, ***P < 0.001

To evaluate the virulence of PmCQ2Δ4555–4580, mice were infected with PmCQ2Δ4555–4580 (8.4 × 10^8^ CFU) by intraperitoneal injection. The survival rates of mice were significantly increased (Fig. 3A), and the bacterial colonization in the mouse lung, liver, and spleen tissues postinfection with PmCQ2Δ4555–4580 was lower than that of PmCQ2 (Fig. 3B-D). Likewise, compared with PmCQ2 (LD_50_ = 0.692 CFU), the LD_50_ of PmCQ2Δ4555–4580 (LD_50_ = 1.941 CFU) was increased by approximately 2.8 × 10^9^ times (Table 1). Thus, these results indicate that the virulence of PmCQ2Δ4555–4580 was strongly attenuated.

**Table 1:** Determination of the LD_50_ in PmCQ2 and PmCQ2Δ4555–4580

PmCQ2	Infection dose (CFU)	75	19	5	2	1
	Euthanized/total mice	8/8	8/8	7/8	6/8	5/8
LD_50_ = 0.692 CFU						
PmCQ2Δ4555–4580	Infection dose (CFU)	7.57 × 10^9^	3.79 × 10^9^	1.89 × 10^9^	9.46 × 10^8^	4.73 × 10^8^
	Euthanized/total mice	8/8	7/8	5/8	0/8	0/8

LD_50_ = 1.941 × 10^9^ CFU

### The immune protection of PmCQ2Δ4555–4580

**Fig. 4:**
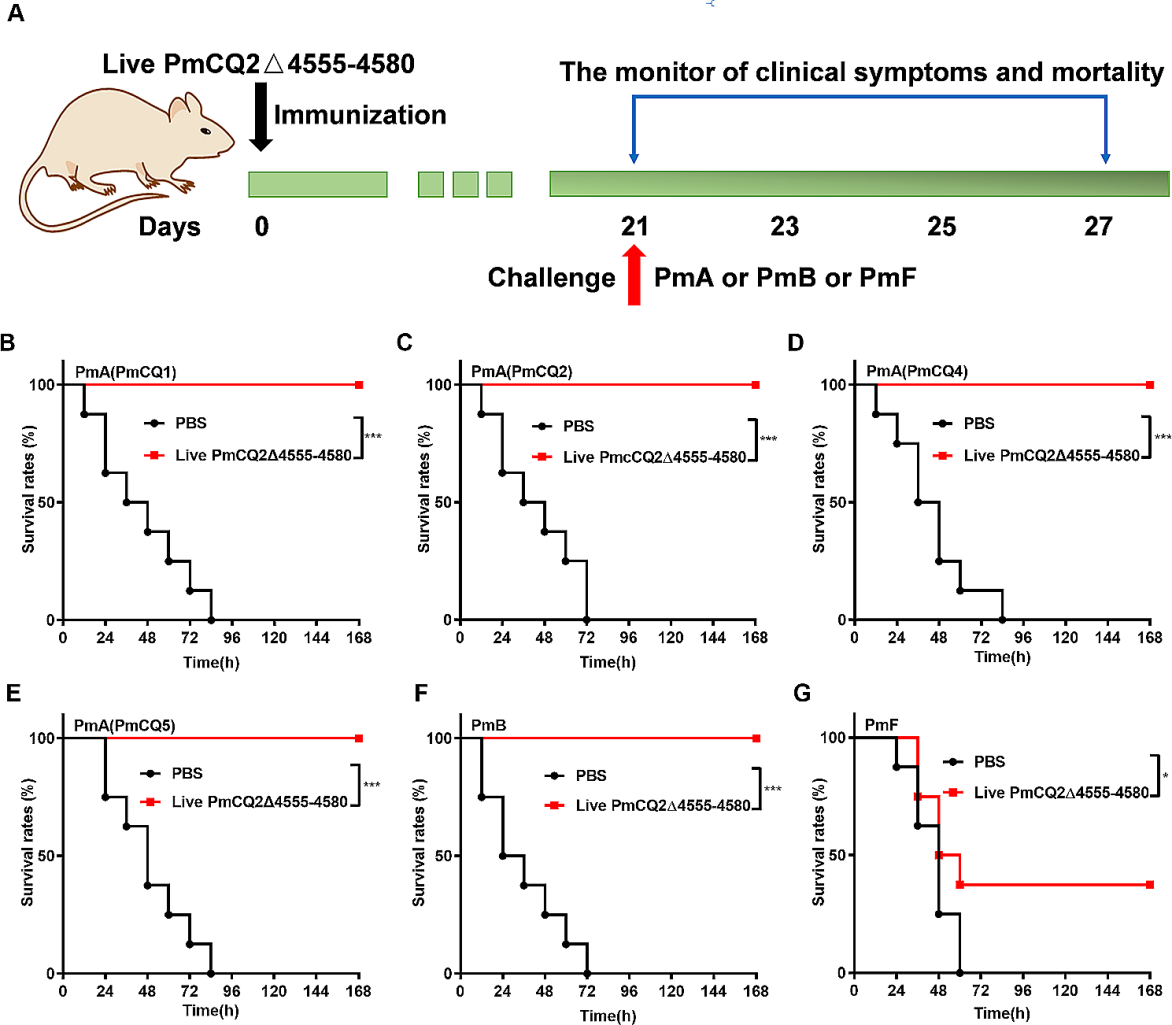
**The immune protection of PmCQ2Δ4555–4580 against Pm.** **A**: Scheme of immunization and infection. **B-E**: The percentage survival curve for immunized and control mice (*n* = 8) following a challenge with PmCQ1 (4.9 × 10^7^ CFU)**(B)**, PmCQ2 (3 × 10^7^ CFU)**(C)**, PmCQ4 (3.8 × 10^7^ CFU)**(D)**, PmCQ5 (5.8 × 10^7^ CFU) **(E)**, PmB (3 × 10^7^ CFU), and PmF (1.0 × 10^8^ CFU). B-G were pooled from two independent experiments with 10 replicates per group

To evaluate the protective efficacy of live PmCQ2Δ4555–4580, mice were immunized with PmCQ2Δ4555–4580 (8.63 × 10^8^ CFU) by intramuscular injection and challenged with PmA (PmCQ1, PmCQ2, PmCQ4 and PmCQ5), PmB and PmF (Fig. 4A). As shown in Supplementary Fig. 2A and B, the serum antibody levels of live PmCQ2Δ4555–4580 immunized mice remained high for at least 77 days. Importantly, mice immunized with live PmCQ2Δ4555–4580 showed excellent tolerance for PmA challenges (100%), including PmCQ1 (4.9 × 10^7^ CFU), PmCQ2 (3.0 × 10^7^ CFU), PmCQ4 (3.8 × 10^7^ CFU), and PmCQ5 (5.8 × 10^7^ CFU), while control mice offered no resistance to PmA challenges and died within one week (Fig. 4B-E). Additionally, the protective effects of live PmCQ2Δ4555–4580 against PmB (3.0 × 10^7^ CFU) and PmF (1.0 × 10^8^ CFU) were 100% and 40%, respectively (Fig. 4F and G). These results indicate that live PmCQ2Δ4555–4580 is a potential vaccine candidate that could provide full protection against PmA and PmB, and moderate protection against PmF infection. Moreover, the protective efficacy of inactivated PmCQ2Δ4555–4580 was also explored, mice immunized with inactivated PmCQ2Δ4555–4580 (5 × 10^8^ CFU) could provide 100% and 87.5% protection against PmA (3.0 × 10^7^ CFU) and PmB (3.0 × 10^7^ CFU) (Supplementary Fig. 3A-C). There were no adverse effects observed (e.g., lesion, fever, and weight loss) after immunization with live or inactivated PmCQ2Δ4555–4580. Together, the above results indicated that PmCQ2Δ4555–4580 is a potential attenuated vaccine candidate for Pm infection.

### The reasons for the increased immune-protective effect of PmCQ2Δ4555–4580 was investigated by bioinformatics analysis

**Fig. 5:**
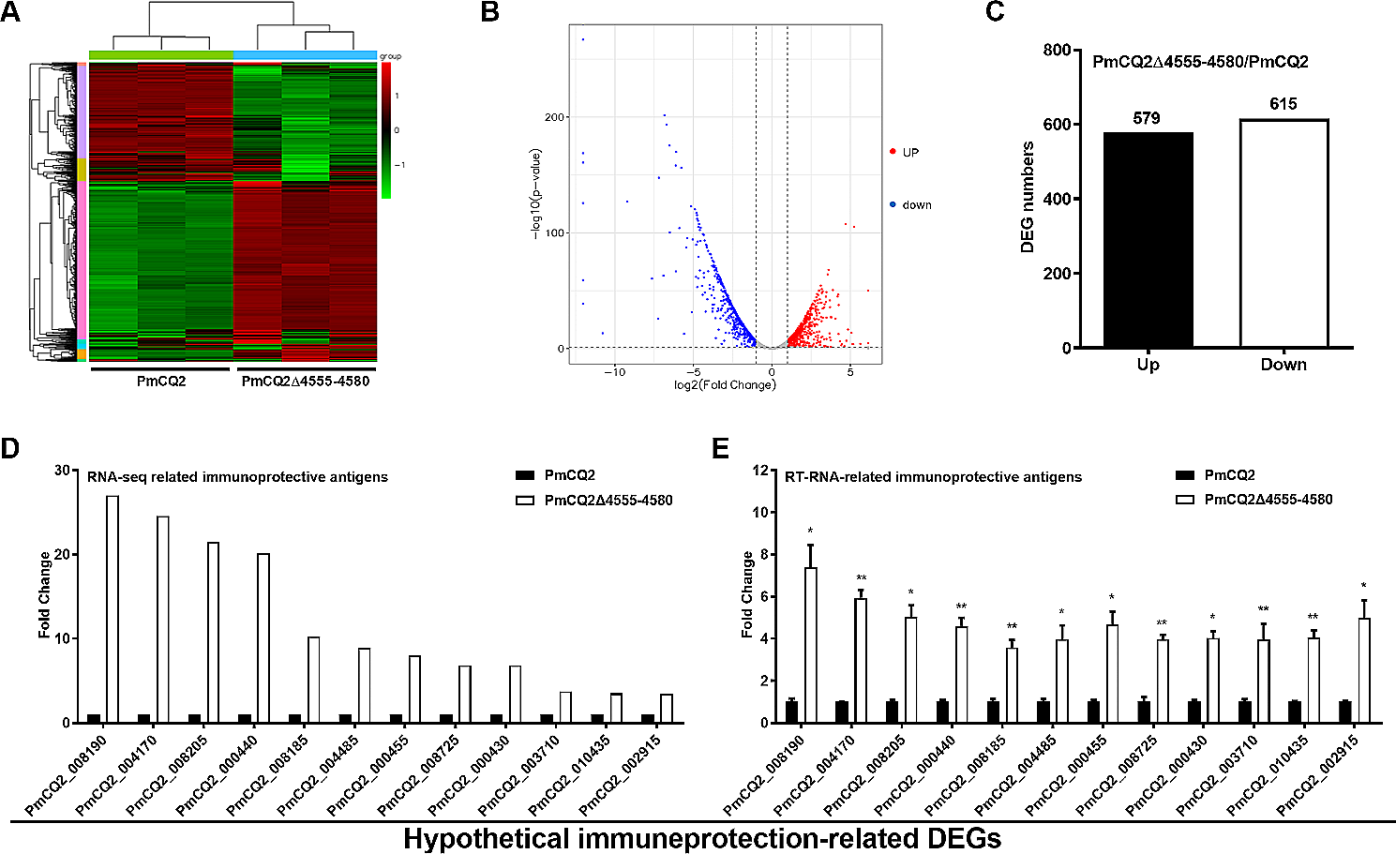
**The expression of hypothetical immune protective proteins.** **A**: Heat-map for clustering of DEGs (FC ≥ 2) in PmCQ2 and PmCQ2Δ4555–4580. **B**: Volcano-map of PmCQ2 and PmCQ2Δ889–894. **C**: The up/down-regulated DEGs of PmCQ2 and PmCQ2Δ4555–4580. **D-E**: Immunoprotective antigens-related DEGs in RNA-seq **(D)** and in RT-qPCR (*n* = 3) **(E)**. E was pooled from two independent experiments with 3 replicates per group, and was analyzed by multiple comparative analysis, and expressed as means ± SD. *P < 0.05, **P < 0.01

To explore why PmCQ2Δ4555–4580 has a powerful protection, the DEGs of PmCQ2 and PmCQ2Δ4555–4580 were compared by transcriptome analysis. A total of 1194 DEGs were observed (Fig. 5A, B), including 579 upregulated and 615 downregulated genes (fold change ≥ 2) (Fig. 5C). Through bioinformatics analysis, including the prediction of signal peptides, transmembrane domains, subcellular localization, and antigen epitopes (Supplementary Table 2), 12 significantly upregulated DEGs were screened (Fig. 5D). Moreover, these DEGs from RNA-sequence analysis were also validated by RT-qPCR analysis (Fig. 5E), and the trends of the two results were consistent. The above results indicate that the expression of immune protection-related proteins was upregulated in PmCQ2Δ4555–4580.

### The protective effect of PmCQ2Δ4555–4580 was related to the high expression of immune-protective related proteins

**Fig. 6:**
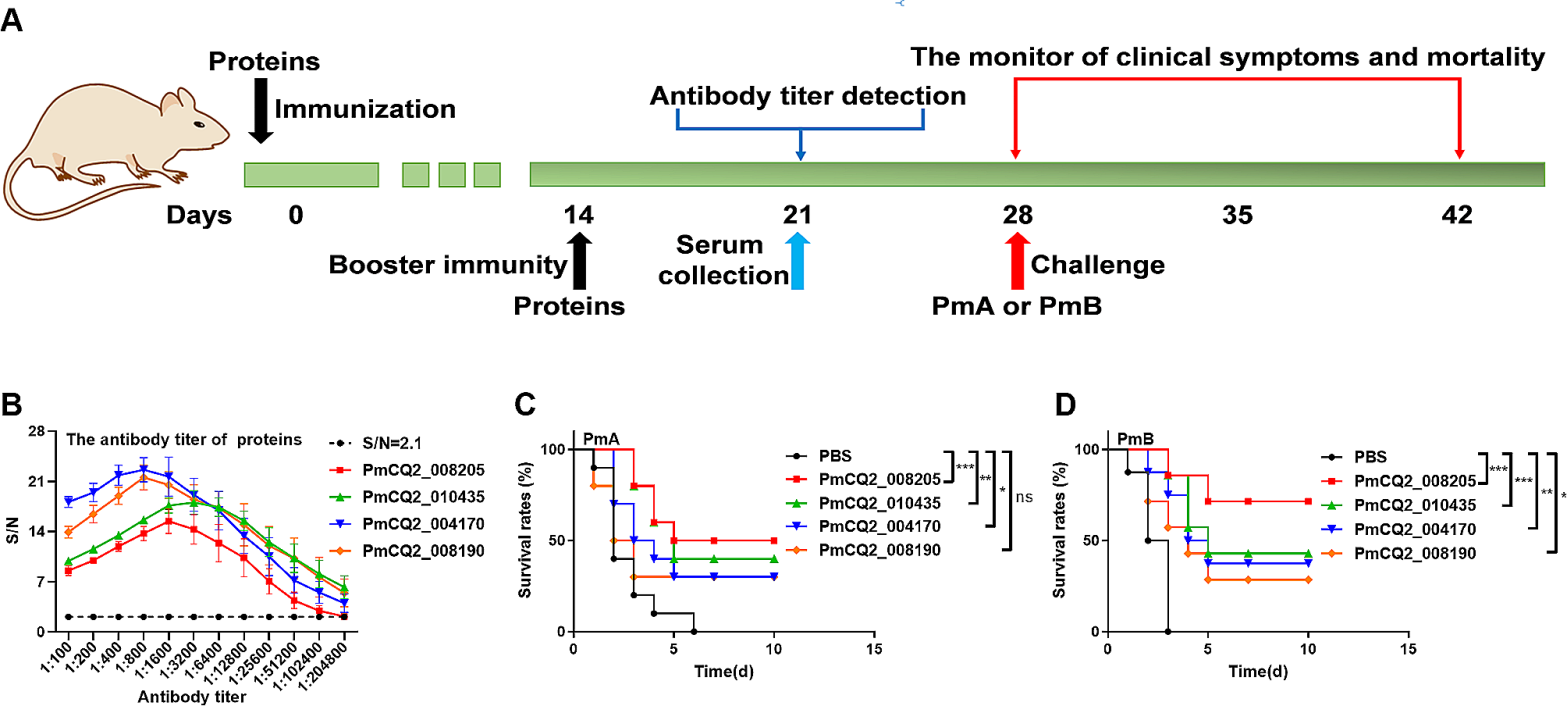
**Immune protection of immune protective proteins against Pm.** **A**: Scheme of immunization and infection. **B**: The antibody titer of immunized (immunoprotective antigens) and control mice (*n* = 8). **C**: The survival curve for immunized and control mice (*n* = 8) following a challenge with PmCQ2 (3 × 10^7^). **D**: The survival curve for immunized and control mice (*n* = 8) following a challenge with PmB (3 × 10^7^ CFU). The data of C and D were pooled from two independent experiments with 10 replicates per group

Given that these upregulated proteins may be involved in the promotion of PmCQ2Δ4555–4580 immune protection, we consequently expressed and purified these proteins (Supplementary Fig. 4A-E) and then immunized mice (Fig. 6A). All proteins could induce mice to produce higher levels of antibodies (Fig. 6B, Supplementary 5A and C). Importantly, all of these proteins have different degrees of immune protective effects in vivo (Supplementary Table 4, Fig. 6C and D, Supplementary Fig. 5B and D). Among them, PmCQ2_008205, PmCQ2_010435, PmCQ2_008190, and PmCQ2_004170 had the best protection against PmA, with protective rates of 50%, 40%, 30%, and 30%, respectively (Fig. 6C). Additionally, the protective rates of PmCQ2_008205, PmCQ2_010435, PmCQ2_008190, and PmCQ2_004170 against PmB were 62.5%, 42.9%, 37.5%, and 28.6%, respectively (Fig. 6D). Collectively, the enhanced immune protection of PmCQ2Δ889–894 was related to the expression of immune protection-related proteins.

## Discussion

Pm is a gram-negative bacterium that causes diseases in poultry, livestock, and humans, mainly featuring by *hemorrhagic septicaemia* and respiratory diseases [16]. PmA usually causes bovine respiratory disease syndrome and pneumonia, resulting in enormous economic losses for the cattle industry [17]. The most effective drugs against bacterial pathogens are antibiotics, but antibiotic resistance and contamination present a great challenge to the prevention and control of Pm infection. To avoid antibiotic-related problems, vaccination is one of the most attractive strategies for controlling infectious diseases. Unfortunately, there is no effective vaccine against PmA infection thus far, and the cross-protection effect of vaccines between different serotypes is poor. Therefore, there is an urgent need to develop safer and more effective vaccines against Pm infection.

Here, a mutant strain with a smaller colony morphology and fewer capsules was obtained, which grows more slowly than the parent strain PmCQ2. Furthermore, the mutant strain was identified as PmCQ2 with 6 genes (PmCQ2_004555, PmCQ2_004560, PmCQ2_004565, PmCQ2_004570, PmCQ2_004575, and PmCQ2_004580) missing via whole-genome resequencing, PCR and RT-qPCR. Additionally, the virulence of PmCQ2Δ4555–4580 was significantly reduced, which was $\sim 2.8 \times {10^9}$-fold lower than that of PmCQ2. Importantly, the bacterial loads in the tissues decreased significantly, which avoids potential biosafety concerns with live attenuated vaccines. As predicted, the PmCQ2-004555 gene encodes the 50 S ribosomal protein L11 methyltransferase, which is important for protein synthesis in bacteria [18]; PmCQ2-004560 encodes tRNA dihydrouridine synthase DusB that is important for dihydrouridine synthesis [19]; PmCQ2-004565 encodes an Fis family transcriptional regulator that is critical for the modulation of virulence factor production and pathogenicity [20]; PmCQ2-004570 encodes phosphorribosylformyl -glycinamidine synthase, which is important for purine [21]; and PmCQ2-004575 encodes a hypothetical protein; PmCQ2-004580 encodes domain of unknown function (DUF) 26-containing protein, plays an important role in signal transduction [22]. Whether the significant reduction in virulence is due to the deletion of PmCQ2-004565, or if multiple genes work together, remains unclear. Thus, the key is to construct deletion strains of each of the six genes to determine which gene is the most important.

Virulence factors contribute to evasion of the immune defenses of Pm in cattle (e.g., capsule, LPS, and OMPs), which ultimately leads to pneumonic Pasteurellosis [7]. The capsular polysaccharide is one of the important virulence factors of Pm [23], and the pathogenicity of the capsule mutants constructed by PmA and PmB are reduced in mice [24, 25]. In this study, capsular synthesis-related genes (*phyA, hyaB, hyaE*, *hexA, hexB, hexC*, and *hexD*) [26], capsule synthesis regulatory genes (*Hfq*, and *Fis*) [20, 27] (Supplementary Fig. 6A-B), and the capsule content were significantly downregulated in PmCQ2Δ4555–4580. LPS is composed of lipid A and core oligosaccharide (OS), which also play an important role in the pathogenic mechanism of Pm [28]. Transcriptome sequencing results clarified that *lpxA*, *lpxB*, *lpxD*, *kdsA*, *lpxB* (lipid A biosynthesis), *gmhA*, and *gmhB* (core OS biosynthetic process) [29, 30] were significantly downregulated in PmCQ2Δ4555–4580 (Supplementary Fig. 6C-D). Additionally, OMPs are also important proteins on the surface of bacteria, such as PM0442 [31]. RNA sequencing analysis showed that OMP-related genes (*OmpA*, *OmpH*, *and PM0442*) were also significantly downregulated in PmCQ2Δ889–894 (Supplementary Fig. 6E-F). The above results indicate that decreased virulence of PmCQ2Δ4555–4580 is involved in regulating the expression of virulence genes such as capsules, LPS, and OMPs. However, specific genes alone or in combination are responsible for reduced virulence, and the regulatory mechanism is being further investigated.

Vaccine immunization is an economical and efficient preventive measure against Pasteurellosis [12]. However, there is no new vaccine with good cross-protection produced in the past few years. Therefore, a vaccine protecting against different serotypes is urgently needed for the prevention of infections due to Pm. Notably, PmCQ2Δ4555–4580 has a 100% immune protection effect against PmA infection. More importantly, the virulence of PmCQ2Δ4555–4580 (LD_50_ = 1.94 × 10^9^ CFU) is approximately 37 times less than that of our previously constructed mutant ΔqseC (LD_50_ = 5.28 × 10^7^ CFU) [32]. Moreover, the serum antibody levels of the PmCQ2Δ4555–4580 immunized group maintained for a long time (at least 77 days). Surprisingly, PmCQ2Δ4555–4580 has a good cross-protection effect against PmB (100%) and PmF (40%). However, more experimental investigations (e.g., rabbits and cattle) are needed before the PmCQ2Δ4555–4580 vaccine can be applied in veterinary clinics.

Bioinformatics is an efficient way to analyze and predict data from genomics, transcriptomics or proteomics. Germano et al. used genomics techniques to identify four potential antigenic proteins for the development of a new generation of leishmaniasis vaccines [33]. Based on the transcriptome sequencing data of PmCQ2 and PmCQ2Δ4555–4580, the potential secretory protein, outer membrane protein, and antigen epitope rich dominant genes were screened from the DEGs with higher changes in multiple and greater expression amounts as candidate protective antigen genes by bioinformatics analysis tools, such as the SignalP-5.0 server for signal peptide prediction, TMHMM-2.0 server for prediction of transmembrane domains, Cello Prediction for prediction of subcellular localization, IEDB and Immuno-medicine Group were used to predict the B-cell sites and possible epitope determinants of candidate genes, and to screen the dominant fragments of candidate genes.

In the study, 12 novel dominant antigen candidate genes were screened according to the above method of bioinformatics analysis. As predicted, the D-galactose-binding periplasmic protein encoded by PmCQ2_004170 serves as a high-affinity receptor for active transport and chemotaxis toward D-galactose [34]; the outer membrane protein assembly factor BamD encoded by PmCQ2_010435 is an essential lipoprotein for cellular processes [35]; aspartate ammonia-lyase encoded by PmCQ2_004485 is needed for bacterial growth in complex medium (e.g., under anaerobic, acid, and iron-limited conditions) in Pm [36]; PmCQ2_008185 encodes a uxu operon regulator; PmCQ2_000430 encodes phage terminase; PmCQ2_008205 encodes an uncharacterized oxidoreductase; PmCQ2_008725, PmCQ2_003710, PmCQ2_008190, and PmCQ2_008725 encode uncharacterized proteins; PmCQ2_000440, PmCQ2_000455, and PmCQ2_002915 encode hypothetical proteins. Notably, the subunit vaccines prepared by these 12 proteins all have immune protective effects, protective efficacy of PmCQ2_008205, PmCQ2_010435, PmCQ2_008190, and PmCQ2_004170 are relatively high (30-50%), but the immune protection of the other eight proteins (PmCQ2_000440, PmCQ2_000430, PmCQ2_003710, PmCQ2_000455, PmCQ2_004485, PmCQ2_002915, PmCQ2_008725, and PmCQ2_008185) was relatively weak (10-25%). These results indicate that these 12 antigenic proteins are related to the protection of PmCQ2∆4555–4580. However, compared to the recently reported antigens of *P. multocida*, such as fur (80%) [13], rTorA, rPrx, and/or rPGAM (60–80%) [37], the protective properties of the 12 antigenic proteins not optimal subunit vaccine candidates. Additionally, the antibody titers do not seem to be fully correlated with protection levels, indicating the immune mechanism of each protein is different, and both humoral and cellular immunity may be involved. Actually, the specific function of other 11 proteins besides PmCQ2_004485 in Pm is still unknown and needs further experimental investigation.

In conclusion, compared with PmCQ2, the mutant strain PmCQ2∆4555–4580 became a low virulence strain with a brand-new phenotype. Importantly, PmCQ2∆4555–4580 has good immune and cross-immune-protective effects in Pm-infected murine models. The increased immune protection of PmCQ2∆4555–4580 may be related to the upregulated immune-protective antigens (e.g., PmCQ2_008205, PmCQ2_010435, PmCQ2_008190, and PmCQ2_004170). Thus, our findings demonstrated that PmCQ2∆4555–4580 is a potential vaccine candidate, providing a new guidance for the prevention of Pm.

## Methods

### Bacterial strains and growth conditions

The highly virulent bovine PmA CQ2 (PmCQ2, GenBank accession number: CP033599) is isolated from the lung of a calf with pneumonia in Chongqing, China [38]. PmCQ2Δ4555–4580 was obtained from PmCQ2 after serially passaging at 42℃. These two strains were streaked on Martin agar plates and incubated for 24 h at 37 ℃, and a single colony was picked and inoculated into 5 mL Martin broth and cultured for 12 h at 37℃ with shaking at 220 r/min.

### Experimental animals and ethics statement

In this study, Female KM mice (7–8 weeks old) purchased from Hunan SJA Laboratory Animal Co., Ltd (Changsha, China) were housed in individually ventilated, pathogen-free cages (temperature at 20–30℃, relative humidity at 50–60%, and lighting cycle at 12 h/day) with free access to food and water. The animal experiments were approved by Chongqing Laboratory Animal Management Committee [License No: SYXK (Yu) XK2019-0003], and were performed strictly in accordance with the guideline of Basel Declaration and recommendations of the Laboratory Animal Ethical Commission of Southwest University to minimize animal sufferings. Finally, the mice were anesthetized by intraperitoneal injection of 100 uL of pentobarbital sodium, and then euthanized by the physical method of cervical spine fracture.

### Whole-genome resequencing of PmCQ2Δ4555–4580

Genomic DNA of PmCQ2 and PmCQ2Δ4555–4580 was obtained using Tiangen DNA extraction kits. The integrity of DNA was detected by 1% agarose gel, and DNA concentration was quantitatively detected by Qubit. Genome resequencing was performed using an Illumina MiSeq platform. The quality of the original sequencing data was evaluated by FastQC (https://www.bioinformatics.babraham.ac.uk/projects/fastqc). Valid sample data were mapped to the reference genome using BWA (https://biobwa.sourceforge.net/), and HaplotypeCaller from GATK (https://gatk.broadinstitute.org/hc/en-us) was used to analyze genotype differences between each sample and the reference genome. Mutations were annotated based on the reference genome by SnpEff (https://pcingola.github.io/SnpEff) software. GO and KEGG pathway enrichment analysis via topGO and cluster profile. PCR verification was performed on the sequencing results with specific primers (Supplementary Table 1).

### Growth conditions of PmCQ2Δ4555–4580 in vitro

PmCQ2 and PmCQ2Δ4555–4580 single colonies were inoculated into 5 mL Martin liquid medium, and cultured in a shaker (220 r/min) at 37 °C for 8 h. Then 1 mL fresh culture was transferred to 100 mL Martin liquid medium, and cultured in a thermal incubator at 220 r/min (37 °C), and the OD_600_ of the bacterial cultures was determined every 2 h using a microplate reader.

### Quantification of hyaluronic acid in the capsule of PmCQ2Δ4555–4580

The content of hyaluronic acids in PmCQ2Δ4555–4580 was measured according to our previous description [31, 39]. Briefly, 100 mL fresh PmCQ2Δ4555–4580 in Martin liquid medium was incubated at 37 °C with 220 r/min for 8 h (logarithmic phase). Then the culture was centrifuged at 7,600×g for 15 min and the supernatant was removed. Next, the bacterial cells were washed twice with PBS, and then incubated for 1 h at 42 °C. The number of bacteria was counted on Martin agar plates before and after incubation at 42 °C. Bacterial solutions were centrifuged, and the supernatant was collected for the detection of capsule content. Then, 10 µL of sample and/or 10 µL of hyaluronic acid standards were added to 90 µL of capsule staining solution (0.2 g/mL Stain all staining solution, 0.06% glacial acetic acid in 50% formamide). Finally, the absorption of OD_640_ was determined by a microplate reader, and the capsule content was calculated. The capsular content of each bacterium (µg /CFU) = Total capsular content(µg)/number of bacteria (CFU). The number of bacteria (CFU) = Average (the number of bacteria before 42℃+the number of bacteria after 42℃).

### Pathogenicity of PmCQ2Δ4555–4580

To determine the virulence of PmCQ2Δ4555–4580, KM mice were infected by intraperitoneal exposure to PmCQ2, or PmCQ2Δ4555–4580, at a dose of 8.4 × 10^8^ CFU in 100 mL. Mice were monitored for 7 days to determine the survival curves, and mice showing severe clinical signs (e.g., depression, accelerated breath, cough, hairiness and lethargy) were considered moribund, and were humanely killed. Lung, liver, and spleen were obtained from the mice (*n* = 10/group) at 4 and 8 h postinfection. Then, bacterial loads were measured as described in a previous study [17].

### Median lethal dose (LD_50_) of PmCQ2Δ4555–4580

The LD_50_ of PmCQ2Δ4555–4580 was measured as described previously [31]. Briefly, mice in the control groups were divided into five groups (8 mice per group), and injected intraperitoneally with 100 µL of various doses of PmCQ2 (75, 19, 5, 2, 1 CFU). Mice in the five experimental groups (8 mice per group) were infected intraperitoneally with 100 µL of various doses of PmCQ2Δ4555–4580 (7.57 × 10^9^, 3.785 × 10^9^, 1.89 × 10^9^, 9.46 × 10^8^, and 4.73 × 10^8^ CFU). Then, mice in all groups were monitored for one week to determine the survival curves, and moribund mice were euthanized humanely. Finally, the LD_50_ of PmCQ2Δ889–894 was calculated using the Bliss method.

### Immune protection of live PmCQ2Δ4555–4580

To explore the role of immune protection of live PmCQ2Δ4555–4580, female KM mice were randomly divided into fourteen groups (*n* = 8/group). The experimental groups of mice were inoculated intramuscularly with 100 µL live PmCQ2Δ4555–4580 (8.63 × 10^8^ CFU), and the control groups were inoculated with 100 µL PBS. Then the mice were intramuscularly injected with 4.9 × 10^7^ CFU PmCQ1 (intramuscular route: LD_50_ = 3.8 × 10^2^ CFU), 3.0 × 10^7^ CFU PmCQ2 (intramuscular route: LD_50_ = 3.4 × 10^3^ CFU), 3.8 × 10^7^ CFU PmCQ4 (intramuscular route: LD_50_ = 2.1 × 10^3^ CFU), 5.8 × 10^7^ CFU PmCQ5 (intramuscular route: LD_50_ = 4.5 × 10^3^ CFU), 3.0 × 10^7^ CFU PmB (intramuscular route: LD_50_ = 5.0 × 10^3^ CFU), and 1.0 × 10^8^ CFU PmF (intramuscular route: LD_50_ = 1.0 × 10^8^ CFU) on day 21 after inoculation. The mice were then monitored for one week, and dying mice were humanely euthanized according to the clinical symptoms.

### Immune protection experiment of inactivated PmCQ2Δ4555–4580

The inactivated PmCQ2 and PmCQ2Δ4555–4580 were prepared as described previously [40]. Briefly, 0.4% formalin was added to the bacterial culture mixture, followed by incubation at 37 °C for 24 h. Next, inactivated PmCQ2 and PmCQ2Δ4555–4580 were diluted with PBS and obtained by mixing with oil (17:3). Then, mice were inoculated intramuscularly with 100 µL of inactivated PmCQ2 and PmCQ2Δ4555–4580 (5 × 10^8^ CFU) on day 0 and day 7, respectively. Next, the mice were injected intramuscularly with PmCQ2 (3 × 10^7^ CFU) and PmB (3.0 × 10^7^ CFU) on day 21 after the first inoculation. The mice were then monitored for one week, and dying mice were humanely euthanized according to the clinical symptoms. Finally, the number of surviving mice in each group was recorded.

### Transcriptome analysis

To explore the details of increased immune protection in PmCQ2Δ4555–4580, fresh bacteria were collected as previously described [31]. Briefly, PmCQ2 (*n* = 3) and PmCQ2Δ4555–4580 (*n* = 3) were obtained and quickly frozen in liquid nitrogen. Then the bacterial samples were sent to the Beijing Genomics Institute (BGI, Shenzhen, China) for transcriptome sequencing and analysis (HiSeq, Illumina). The data have been deposited in NCBI’s Sequence Read Archive (SRA) database and the accession number is PRJNA998556.

### Real-time-quantitative-PCR (RT-qPCR)

Total RNA of the bacterial samples was extracted by RNA Kit (TIANGEN) based on the manufacturer’s instructions. Next, extracted RNA was reverse transcribed to cDNA by Reverse Transcription Master Mix (US Everbright Inc) according to manufacturer’s recommendations. Then, RT-qPCR was performed via SYBR Green on a CFX96 instrument (Bio-Rad). Relative expression of genes was calculated using the 2^−ΔΔCt^ method with β-actin for reference. Primer sequences for RT-qPCR are listed in Supplementary Table 1.

### Screening of protective antigens by bioinformatics analysis

To explore the reasons for the enhanced immunoprotective effect of PmCQ2Δ4555–4580, the potential secretory protein, outer membrane protein, and antigen epitope rich dominant genes were screened from the differentially expressed genes (DEGs) with higher changes in multiple and greater expression amounts as candidate protective antigen genes by bioinformatics analysis tools, such as SignalP-5.0 server (https://services.healthtech.dtu.dk/services/SignalP-5.0) for signal peptide prediction, the TMHMM-2.0 server (https://services.healthtech.dtu.dk/services/TMHMM-2.0) for prediction of transmembrane domains, and Cello Prediction (http://www.csbio.sjtu.edu.cn/bioinf/Cell-PLoc-2/) for prediction of subcellular localization. IEDB (http://www.iedb.org/) and Immunomedicine Group (http://imed.med.ucm.es/Tools/antigenic.pl) were used to predict the B-cell sites and possible epitope determinants of candidate genes. Based on the above methods, 12 hypothetical protective antigens were screened (Supplementary Table 2).

### Expression and purification of immunoprotection-related proteins

The results of whole-genome resequencing showed that the sequences of target proteins in the wild-type and mutant strains were consistent. Thus, the specific primers (Supplementary Table 3) for target genes are designed based on PmCQ2 genomic data in Gene Bank. Then, the target genes were amplified from the genomic DNA of PmCQ2Δ4555–4580. Next, the amplified products were inserted into pET28a plasmids (Takara Biotechnology Co., Ltd.), and induced with 0.5 mM isopropyl β-D-1-thiogalactopyranoside (Sigma-Aldrich; Merck KGaA) for expression in *E. coli* BL21 (DE3, Takara Biotechnology Co., Ltd.). In accordance with previously described protocols [31, 38], the recombinant proteins were purified using Ni-NTA Superflow cartridges (Qiagen GmbH, Hilden, Germany) and characterized by SDS-PAGE. Then, the concentrations of proteins were determined by a Pierce™ BCA Protein Assay Kit (Thermo Scientific).

### Immune protection of candidate proteins

A total of 168 female KM mice were equally divided into 21 groups (*n* = 8/group). Experimental group mice were subcutaneously inoculated with the 100 µg of recombinant proteins mixed in Freund’s complete adjuvant (1:4), and control group mice were inoculated with equal amounts of antigen-free placebo (PBS and Freund’s complete adjuvant mixture). Enhanced immunization was performed on the 14th day after initial inoculation, and mice were immunized subcutaneously with 100 µg/dose proteins or PBS in Freund’s incomplete adjuvant. After 7 days of the second immunization, whole blood was collected by tail tip sampling, and centrifuged serum was frozen for later use. Then the mice were intramuscularly injected with PmCQ2 (3 × 10^7^ CFU) and PmB (3 × 10^7^ CFU) on the 14th day after the second immunization.

### Antibody levels of PmCQ2Δ4555–4580

Antibody levels of PmCQ2Δ4555–4580 were determined as the method described previously [32]. Briefly, total bacterial cell proteins of PmCQ2Δ4555–4580 were prepared by using ultrasound pyrolysis, and the proteins concentration was measured by Bradford method. Each well of 96-well ELISA plates was coated with 1 µg protein in 100 µL carbonate buffer (0.05 M, pH9.0) at 4 °C overnight. The next day, the plates were washed 5 times with PBST (PBS containing 0.05% Tween-20), and then treated with blocking buffer (5% skim milk in PBST) at 37 °C for 1 h. Then, the sera were serially diluted in twofold increments in 96-well plates and incubated at 37 °C for 1 h. Next, 100 µL of HRP-conjugated goat anti-mouse IgG (H + L) antibody (Sigma; diluted at 1: 10 000) was added and incubated for 1 h at 37 °C. Then, 100 µL of TMB were added for 10 min (Beyotime biotechnology, China) and stopped by the addition of 2 M H2SO4, before the absorbance quantification at OD_450_ was done. When the ratio of the positive value (P) of the maximum dilution multiple sera of immunized mice to the negative value (N) of sera of non-immunized mice is greater than 2.1 (P/*N* > 2.1), the maximum dilution ratio is the serum antibody titer.

### Antibody levels of recombinant proteins

According to a previous study [32], ELISA was performed to determine the serum antibodies levels of recombinant proteins. In brief, the purified protein sample was diluted with the coating solution to a final concentration of 0.01 µg/µL, and added to 96-well microplates (100 µL/well) incubating at 4 °C overnight. The plates were washed three times with 0.05% PBST, and blocked with 200 µL sealing fluid (2% skim milk) at 37 °C for 1 h. The serum (100 µL/well) collected from mice after 7 days of immunization was used as the primary antibody and incubated at 37 °C for 1 h. Then, horseradish peroxidase-conjugated sheep anti-mouse antibodies in blocking buffer were added to each well and incubated continuously at 37 °C for 1 h. Then, 100 µL TMB substrate solution was added to each well and incubated in the dark for 10 min at room temperature. Finally, the reaction was stopped by adding 100 µL of stop buffer (2 N sulfuric acid) and absorbance values were recorded at OD_450_.

### Statistical analysis

All data are expressed as the means ± standard deviations (SD). All statistical analyses were performed using GraphPad Prism software. The survival rates of the mice were evaluated using Kaplan‒Meier analysis (Prism 6.0). All the other data between two groups were evaluated using unpaired, two-tailed Student’s t test (Prism 6.0). Data among more than two groups were analyzed by one-way ANOVA followed by Dunnett’s multiple comparisons test (Prism 6.0). Significant differences were considered at *p* < 0.05 (∗*p* < 0.05, ∗∗*p* < 0.01, ∗∗∗*p* < 0.001, ∗∗∗∗*p* < 0.0001).

### Electronic supplementary material

Below is the link to the electronic supplementary material.

Supplementary Material 1

Supplementary Material 2

Supplementary Material 3

Supplementary Material 4

Supplementary Material 5

Supplementary Material 6

Supplementary Material 7

Supplementary Material 8

Supplementary Material 9

Supplementary Material 10

Supplementary Material 11

Supplementary Material 12

## Data Availability

Raw sequencing data in this study have been deposited to NCBI’s Sequence Read Archive (SRA) database and the accession number is PRJNA998556 (https://dataview.ncbi.nlm.nih.gov/object/PRJNA998556). The original PCR and SDS-PAGE pictures are available in Supplementary material. All data generated or analyzed during this study are available from the corresponding author by request.

## Abbreviations

Pm: *Pasteurella multocida*
LD50: Median lethal dose
CFU: Colony-forming units
RNA-seq: RNA sequencing
SRA: Sequence read archive
PCR: Polymerase chain reaction
DEGs: Differentially expressed genes
RT-qPCR: Real-time-quantitative-PCR
