# Supplementary figures and images for: Attenuated vaccine PmCQ2Δ4555–4580 effectively protects mice against Pasteurella multocida infection
Source: BMC Vet Res. 2024 Mar 9;20:94. doi: 10.1186/s12917-024-03948-6 (PMC10924365; doi:10.1186/s12917-024-03948-6)

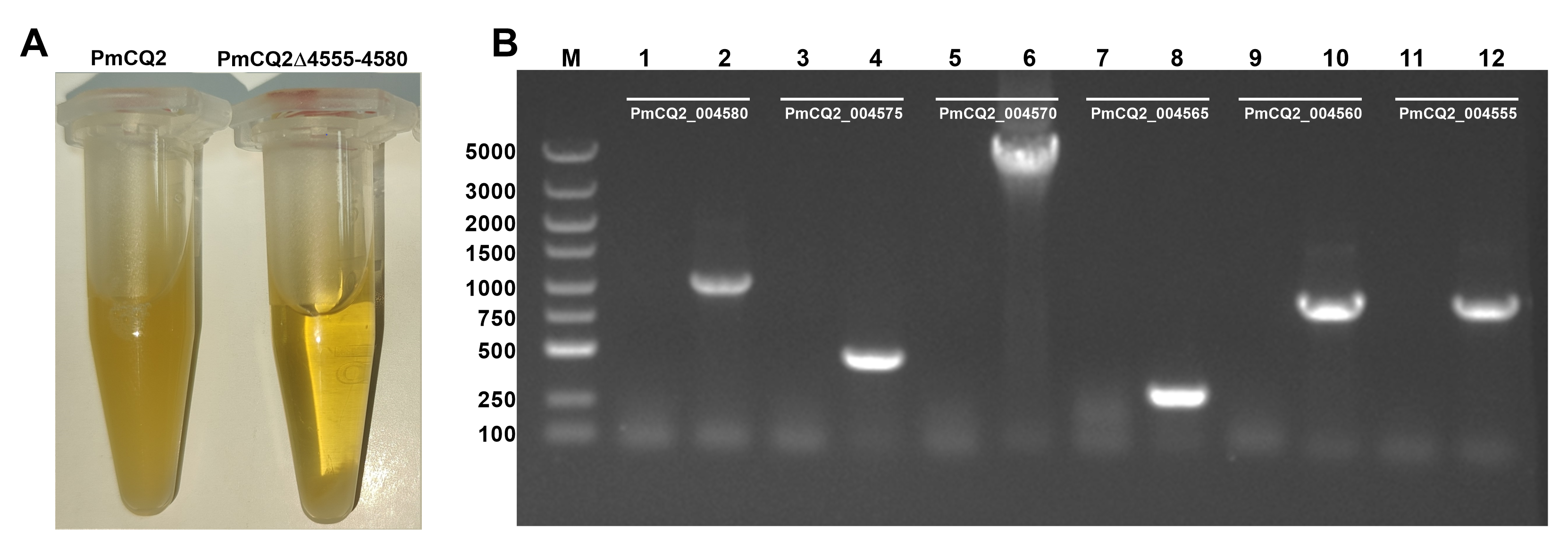

Supplement: Supplementary file 1 — Supplementary Material 1 [file 12917_2024_3948_MOESM1_ESM.tif]

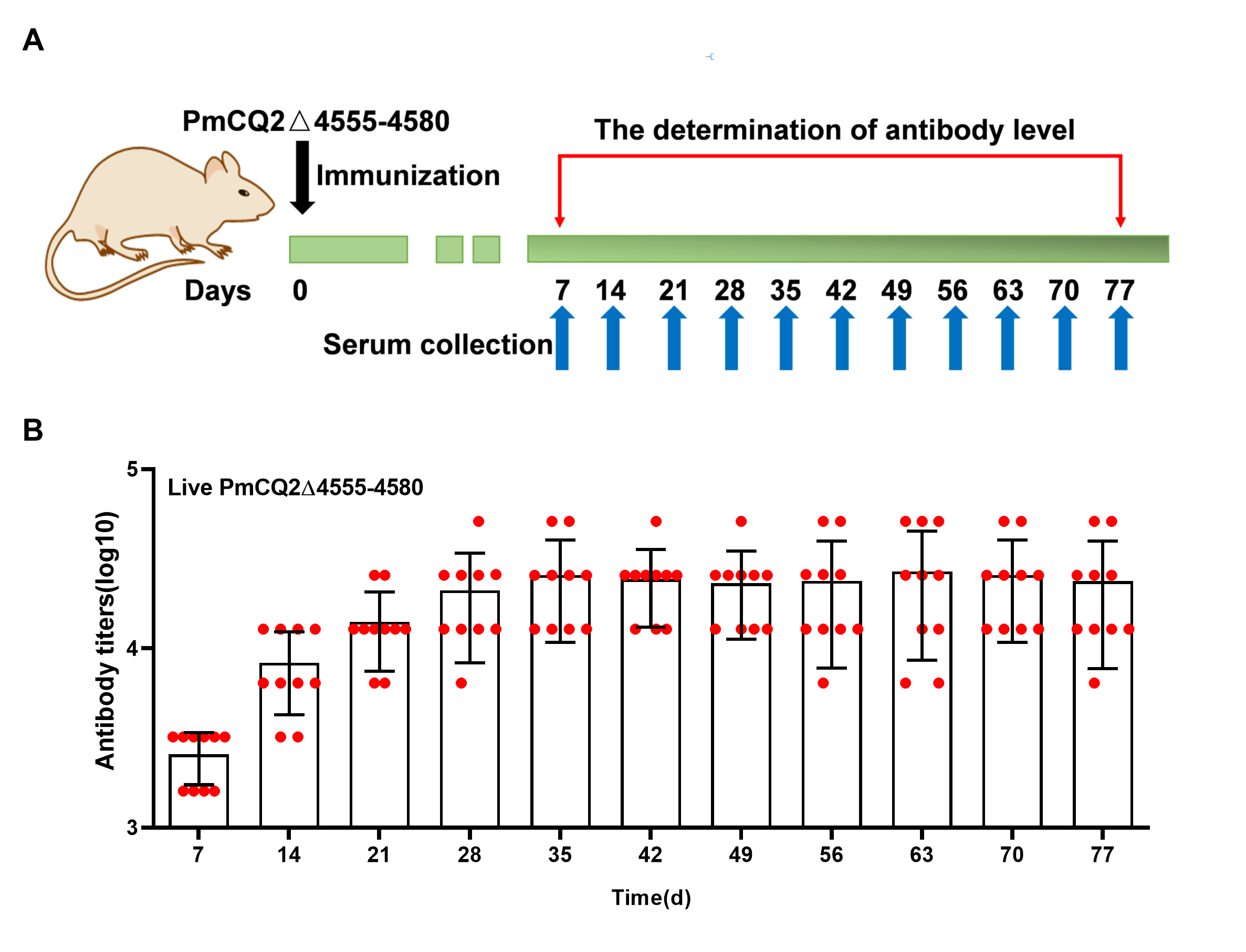

Supplement: Supplementary file 2 — Supplementary Material 2 [file 12917_2024_3948_MOESM2_ESM.tif]

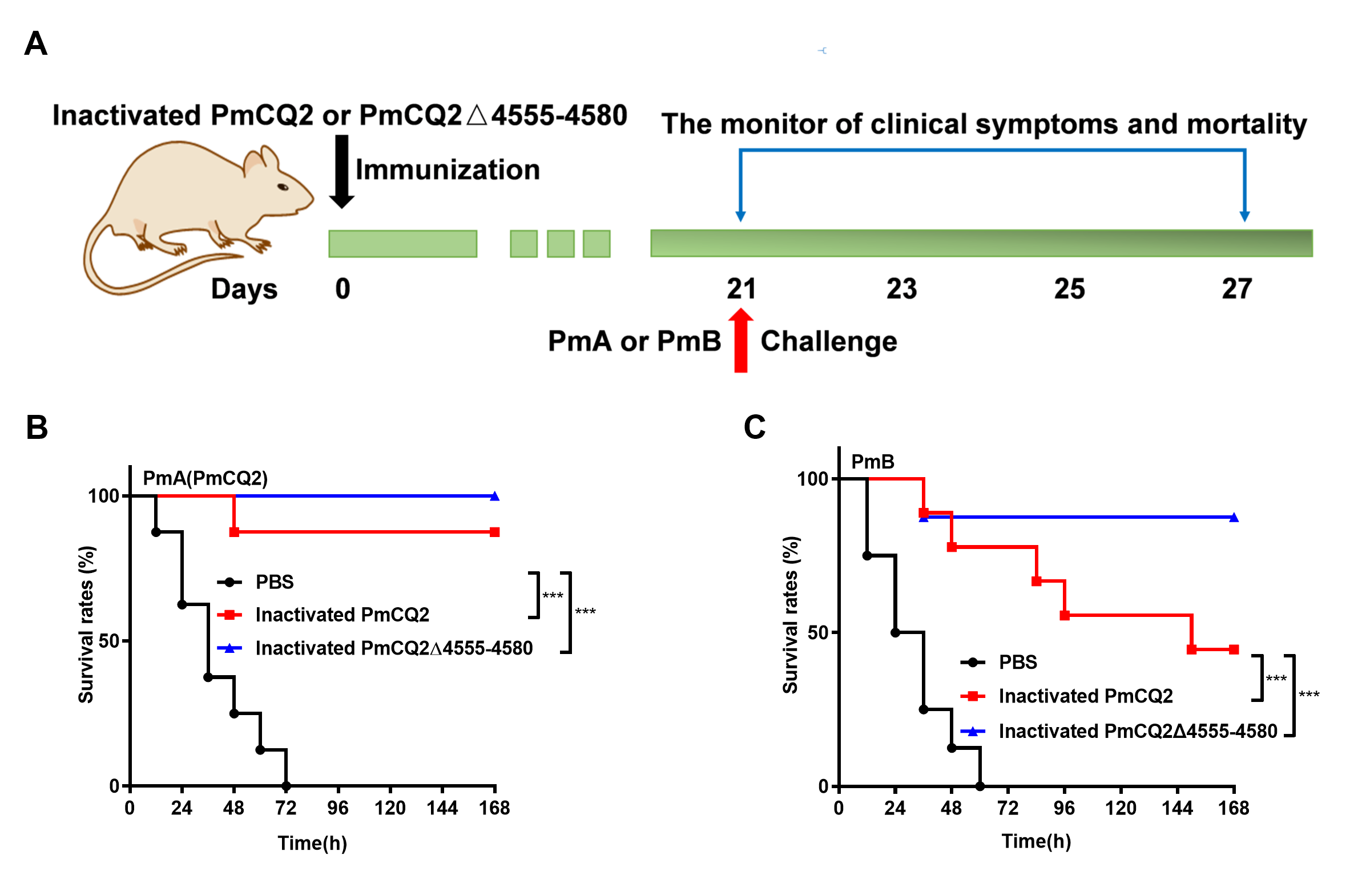

Supplement: Supplementary file 3 — Supplementary Material 3 [file 12917_2024_3948_MOESM3_ESM.tif]

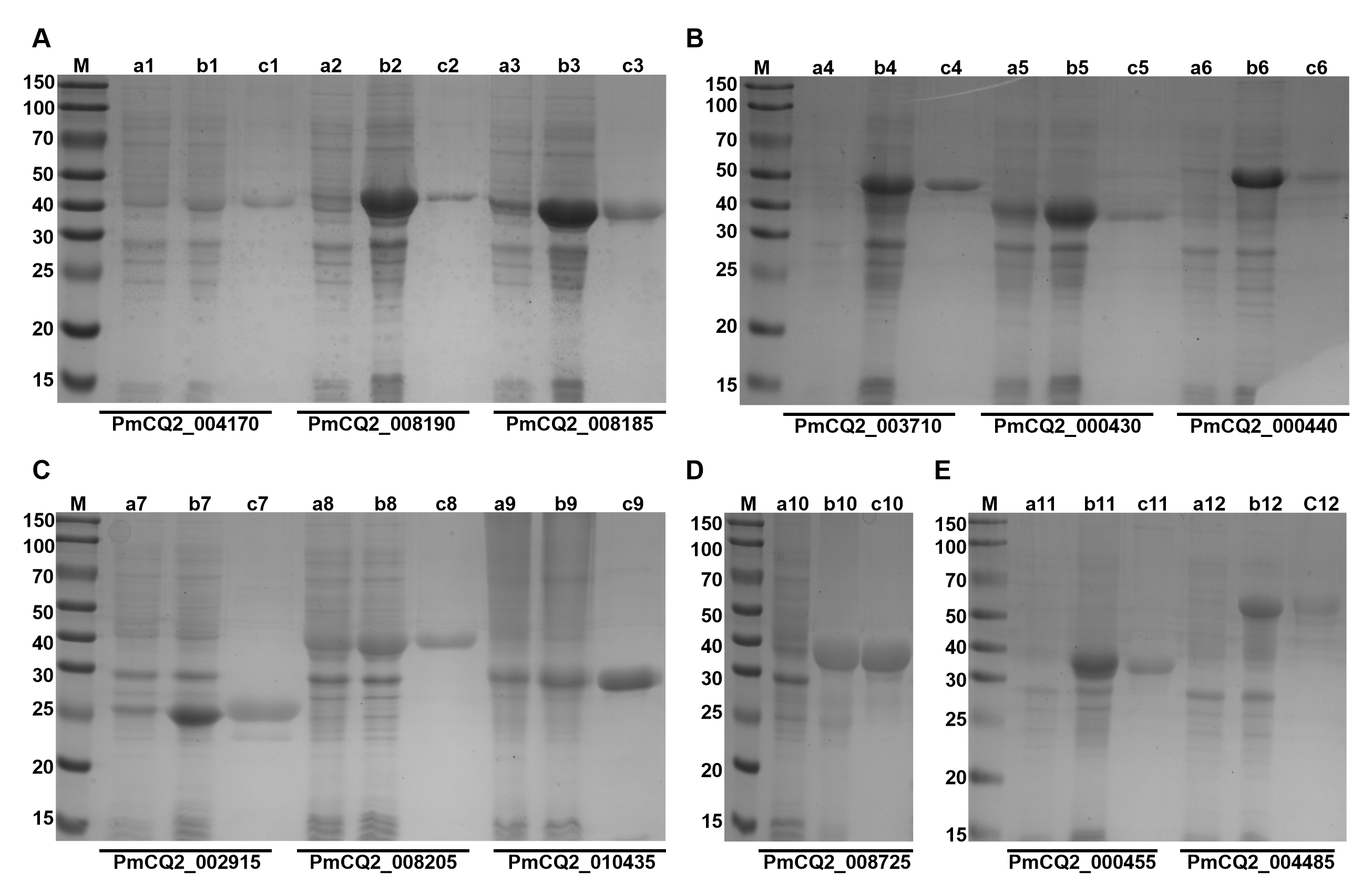

Supplement: Supplementary file 4 — Supplementary Material 4 [file 12917_2024_3948_MOESM4_ESM.tif]

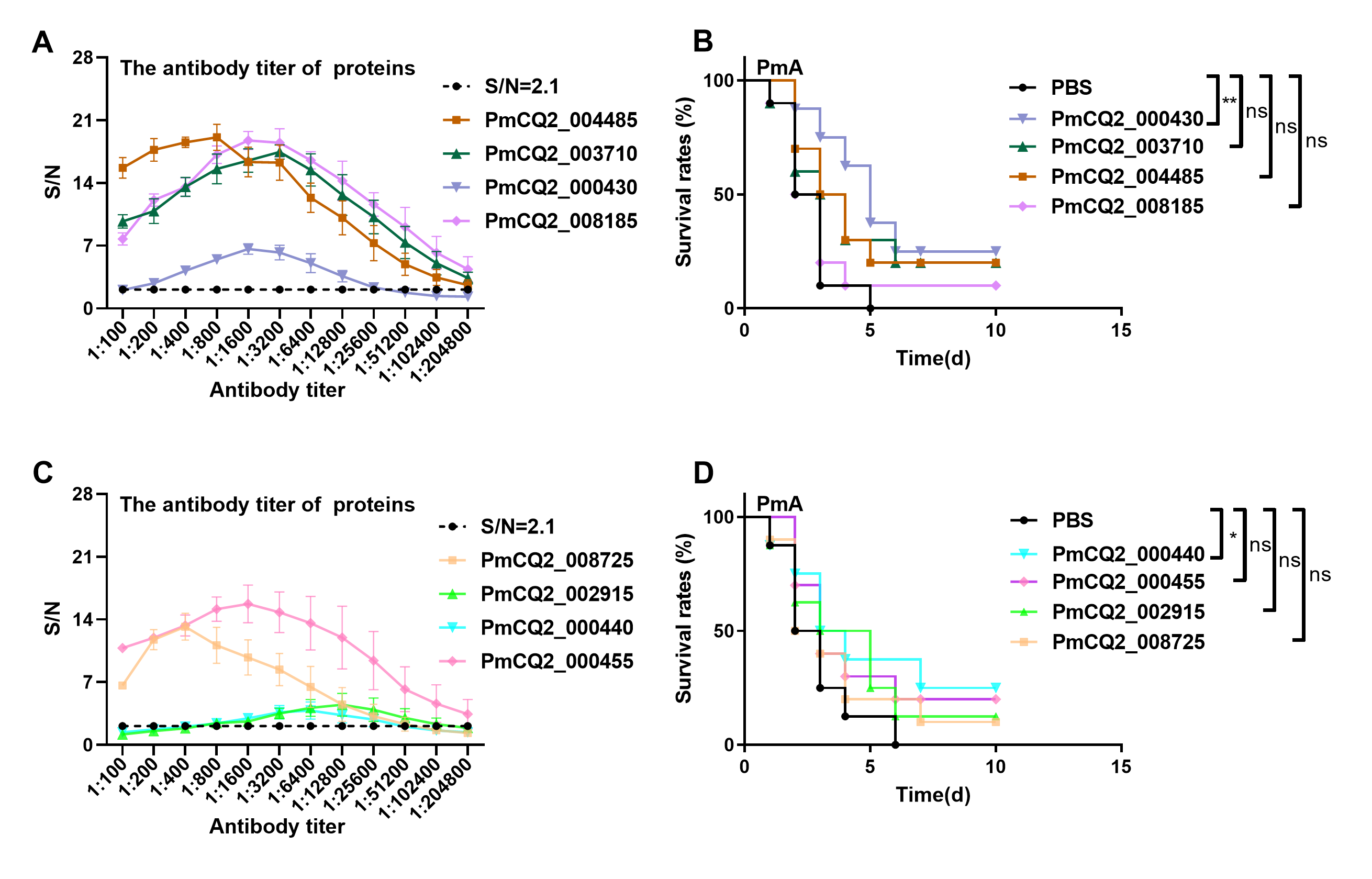

Supplement: Supplementary file 5 — Supplementary Material 5 [file 12917_2024_3948_MOESM5_ESM.tif]

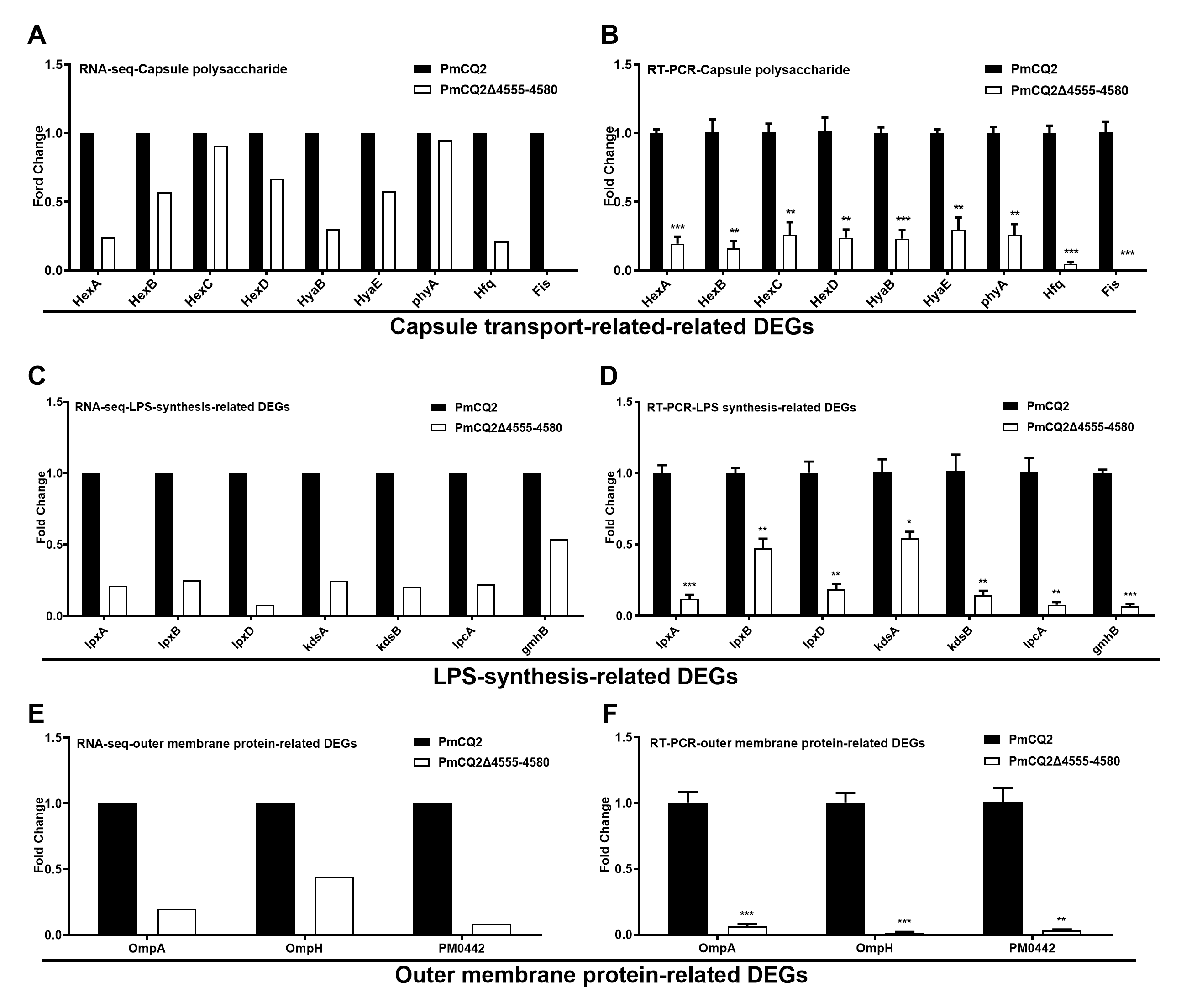

Supplement: Supplementary file 6 — Supplementary Material 6 [file 12917_2024_3948_MOESM6_ESM.tif]
