# Supplementary material for: Attenuated vaccine PmCQ2Δ4555–4580 effectively protects mice against Pasteurella multocida infection
Source: BMC Vet Res. 2024 Mar 9;20:94. doi: 10.1186/s12917-024-03948-6 (PMC10924365; doi:10.1186/s12917-024-03948-6)
Supplement: Supplementary file 7 — Supplementary Material 7 [file 12917_2024_3948_MOESM7_ESM.docx]

**Supplementary Table 1. The primers sequences used in this study**

| **Gene ID** | **Primers（5，→3，）** | **Product (bp)** |
| --- | --- | --- |
| KMT1 | F:TAGATCTCTGGTTGGGGGATTT | 353 |
|  | R:TACGAATAACTAATGAAATCAACA |  |
| CapA | F:TGCCAAAATCGCAGTGAG | 1044 |
|  | R:TTGCCATCATTGTCAGTG |  |
| PmCQ2-004580 | F:CATTTGTCTTGGAACTTGT | 1239 |
|  | R:AAGTGATGATTGCGATGATG |  |
| PmCQ2-004575 | F:AGATAGCGAAGAGTTTAACG | 573 |
|  | R:TAGTATTGTAGAGCGGATTT |  |
| PmCQ2-004570 | F:AGCCAATCTTCCCGTGAG | 3894 |
|  | R:TTAGAAACCGTGCGAAATAC |  |
| PmCQ2-004565 | F:CTAATACACAAGCAGAACAG | 300 |
|  | R:ATACGGCACCATTCTTCG |  |
| PmCQ2-004560 | F:TGTTCTTTGGGATCAGTAAT | 1005 |
|  | R:ATTTTTCTTGCTCCTATGGC |  |
| PmCQ2-004555 | F:TGTTCTTTGGGATCAGTAAT | 882 |
|  | R:ATTTTTCTTGCTCCTATGGC |  |
| HexA-q | F: CAAGGTAGCTTAACGGGTATGG | 105 |
|  | R: ACCTAGCTCAGAGAACTCTTCA |  |
| HexB-q | F: TCAGAGTGCTTGACACGTTAAT | 117 |
|  | R: CCTGTGGCATAGGTATCCAATC |  |
| HexC-q | F: GGCATTGCTACGCTCAATATC | 106 |
|  | R: GCTCATTGAGGCGATTGATAAG |  |
| HexD-q | F: CTCACGACATTACCCGAACAA | 129 |
|  | R: GGAGGATAACGCATGAGCAATA |  |
| HyaB-q | F: GGGTAAGAATCGGTGCAGATAG | 100 |
|  | R: GCACCGAATGTCAAAGGTTTATT |  |
| HyaE-q | F: CCAAGAAGAATGTCCACTGTCTA | 102 |
|  | R: AGATCAGCATGCGACTGTAAT |  |
| phyaA-q | F: CCTCTAATCGGGAACGAGATTG | 99 |
|  | R: GTTCAGTTGGGTTAGGGTCTAAT |  |
| Hfq-q | F: CGTCGTGAACGTATCCCTGT | 151 |
|  | R: GCGCAGGAACAACAGTTGAA |  |
| Fis-q | F: TCGCAGTTAGATGGCCAAGA | 122 |
|  | R: ATTGTCGCTGCACGAGTTTG |  |
| lpxD-q | F: CGAAGAGTACACGCCAGGTT | 188 |
|  | R: TCCATATTGGGACCGGGACT |  |
| lpxA-q | F: ATCGCTGATTCTTTTGCCGC | 190 |
|  | R: CTTATGTGATGGCGCAAGGC |  |
| lpxB-q | F: AAGGTTGCTTCTGCGGCTAT | 78 |
|  | R:GCAAGTCGCGCCAGAATTAG |  |
| kdsA-q | F: ATTCCCCATTTGACCGGGAC | 88 |
|  | R: AACCGATTTAGTGGCAGCGA |  |
| kdsB-q | F: GTGCAACTGTCGCAACTTGT | 128 |
|  | R: ACCTGGGAAACCTTTAGCGG |  |
| LpcA-q | F: GACCGACAGCTTCCACGTAA | 196 |
|  | R: GGGAAAGTGCTTTCTTGCGG |  |
| gmhB-q | F: ATCAGTCAGGTATTGCGCGT | 105 |
|  | R: TGCCATCAAGATCAACGCCT |  |
| OmpH-q | F: TGCTGGCAAACAAGCATCAC | 96 |
|  | R: CCTGCTTCAAGTGCGAAACC |  |
| OmpA-q | F: CGTGACCTAACACAGCAGGT | 190 |
|  | R: AATGCAACACCAGCAACGAC |  |
| PM0442-q | F: TCCGCTGCTTTTTCTGCAAC | 84 |
|  | R: TAAAGCTGCTACGGCGACAA |  |
| PmCQ2-004555q | F: AAACACAATGCGGTCGTTGG | 103 |
|  | R: GTCCAAGTTGGCGTGAGGTA |  |
| PmCQ2-004560q | F: ATCCGTGGCTTTTCCGAGTT | 141 |
|  | R: GACATCCTTTCTCCGCACCA |  |
| PmCQ2-004565q | F: ATTGTCGCTGCACGAGTTTG | 122 |
|  | R: TCGCAGTTAGATGGCCAAGA |  |
| PmCQ2-004570q | F:CGAGTTCTGGCATCGCATTG | 86 |
|  | R:TTGCTGGCAGTTAGGTGAGG |  |
| PmCQ2-004575q | F:GCGCCAAAACGGGAGAATTT | 108 |
|  | R:TGTGGCCAAGTCTAATGTTGT |  |
| PmCQ2-004580q | F:GCGCCAAAACGGGAGAATTT | 158 |
|  | R:TGTGGCCAAGTCTAATGTTGT |  |
| 16sRNA-q | F:ACGCTGGCGGCAGGCTTAAC | 101 |
|  | R:ATTCCCAAGCATTACTCACCCGTCC |  |
