# Supplementary material for: Attenuated vaccine PmCQ2Δ4555–4580 effectively protects mice against Pasteurella multocida infection
Source: BMC Vet Res. 2024 Mar 9;20:94. doi: 10.1186/s12917-024-03948-6 (PMC10924365; doi:10.1186/s12917-024-03948-6)
Supplement: Supplementary file 8 — Supplementary Material 8 [file 12917_2024_3948_MOESM8_ESM.docx]

**Supplementary Table 2. Bioinformatics analysis of protective antigens**

| Protein ID | Description | Subcellular localization | Signal peptide (Yes/No) | Transmembrane (Yes/No) |
| --- | --- | --- | --- | --- |
| PmCQ2_008205 | Uncharacterized oxidoreductase | Extrecellar | N | N |
| PmCQ2_008190 | Uncharacterized protein YiiZ | Outer membrane | Y | Y |
| PmCQ2_010435 | Outer membrane protein assembly factor BamD | Outer membrane | Y | N |
| PmCQ2_004170 | D-galactose-binding periplasmic protein | Periplasmic | Y | Y |
| PmCQ2_003710 | Uncharacterized protein | Periplasmic | Y | N |
| PmCQ2_000430 | Phage terminase | Periplasmic | N | N |
| PmCQ2_002915 | Hypothetical protein UPF0319 protein | Periplasmic | Y | N |
| PmCQ2_000455 | hypothetical protein | Periplasmic | N | N |
| PmCQ2_008725 | Uncharacterized protein | Periplasmic | Y | Y |
| PmCQ2_004485 | Aspartate ammonia-lyase | Periplasmic | N | N |
| PmCQ2_008185 | Uxu operon regulator | Periplasmic | N | N |
| PmCQ2_000440 | hypothetical protein PMCN06_2090 | Periplasmic | N | N |
