# Supplementary material for: Attenuated vaccine PmCQ2Δ4555–4580 effectively protects mice against Pasteurella multocida infection
Source: BMC Vet Res. 2024 Mar 9;20:94. doi: 10.1186/s12917-024-03948-6 (PMC10924365; doi:10.1186/s12917-024-03948-6)
Supplement: Supplementary file 9 — Supplementary Material 9 [file 12917_2024_3948_MOESM9_ESM.docx]

**Supplementary Table 3. The primers of hypothetical immune protective proteins**

| **Gene ID** | **Primers（5，→3，）** | | **Product** | **Protein** |
| --- | --- | --- | --- | --- |
| PmCQ2_008205 | | F:GGCCATGGCTGATATC-GGATCC-gttatcgtcattactggcg | 810bp | 29.7 kDa |
|  |  | R:CTCGAGTGCGGCCGCAAGCTT-ttacacaccgctataagc |  |  |
| PmCQ2_008190 | | F:GGCCATGGCTGATATC-GGATCC-tttaatgcgttagcgagc | 882bp | 32.34 kDa |
|  |  | R:CTCGAGTGCGGCCGCAAGCTT-ctgcagtttacggatctc |  |  |
| PmCQ2_010435 | | F:GGCCATGGCTGATATC-GGATCC-aaagatgttgaacagcgtcc | 705bp | 25.85 kDa |
|  |  | R:CTCGAGTGCGGCCGCAAGCTT-taataagccttcaggctcc |  |  |
| PmCQ2_004170 | | F:GGCCATGGCTGATATC-GGATCC-aatgaccaagtggatggttt | 768bp | 28.16 kDa |
|  |  | R:CTCGAGTGCGGCCGCAAGCTT-ctcgtttaagttatccgc |  |  |
| PmCQ2_000455 | | F:GGCCATGGCTGATATC-GGATCC-ggcaaattctcacaaaagct | 738bp | 27.06 kDa |
|  |  | R:CTCGAGTGCGGCCGCAAGCTT-tgtaattccatcctgcatag |  |  |
| PmCQ2_008725 | | F:GGCCATGGCTGATATC-GGATCC-aatgaaaaactcattg | 918bp | 33.66 kDa |
|  |  | R:CTCGAGTGCGGCCGCAAGCTT-ttattgacgtccttgg |  |  |
| PmCQ2_004485 | | F:GGCCATGGCTGATATC-GGATCC-gtggatttactcggagaacg | 1386bp | 50.82 kDa |
|  |  | R:CTCGAGTGCGGCCGCAAGCTT-cttcgctttataggttgg |  |  |
| PmCQ2_000440 | | F:GGCCATGGCTGATATC-GGATCC-tatgacgcctcactaagacg | 1488bp | 54.56 kDa |
|  |  | R::CTCGAGTGCGGCCGCAAGCTT-cttcatcttctcgttaagtc |  |  |
| PmCQ2_003710 | | F:GGCCATGGCTGATATC-GGATCC-gctactgggttactggcttc | 966bp | 35.42 kDa |
|  |  | R::CTCGAGTGCGGCCGCAAGCTT-ccatttaccaatcaagcg |  |  |
| PmCQ2_000430 | | F:GGCCATGGCTGATATC-GGATCC-ttcagagcttatgttgcagg | 1206bp | 44.22 kDa |
|  |  | R:CTCGAGTGCGGCCGCAAGCTT-tgaggactgtctagaaattgg |  |  |
| PmCQ2_008185 | | F:GGCCATGGCTGATATC-GGATCC-attggcgagcgattacct | 663bp | 24.31 kDa |
|  |  | R:CTCGAGTGCGGCCGCAAGCTT-tattccaacaacaacagga |  |  |
| PmCQ2_002915 | | F:GGCCATGGCTGATATC-GGATCC-gattttcttgccattgatgg | 561bp | 20.57 kDa |
|  |  | R:CTCGAGTGCGGCCGCAAGCTT-ttgacctttggcccaactta |  |  |
