# Supplementary material for: Attenuated vaccine PmCQ2Δ4555–4580 effectively protects mice against Pasteurella multocida infection
Source: BMC Vet Res. 2024 Mar 9;20:94. doi: 10.1186/s12917-024-03948-6 (PMC10924365; doi:10.1186/s12917-024-03948-6)

Supplementary Original data 1 to 8

Original result **Result in manuscript**

Supplementary Original data 1: Figure2A

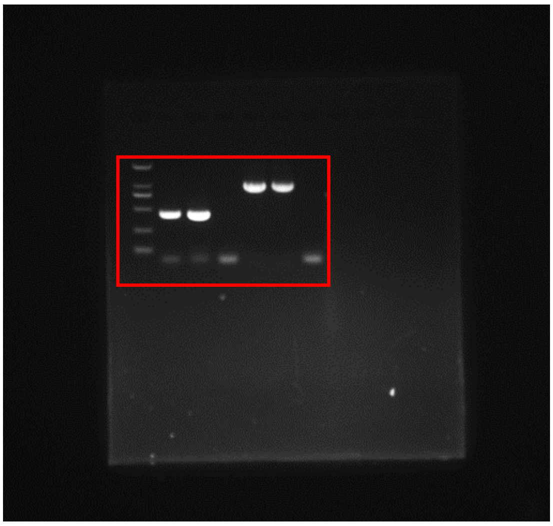

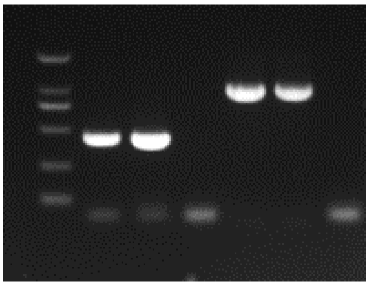

Supplementary Original data 2: Figure2C

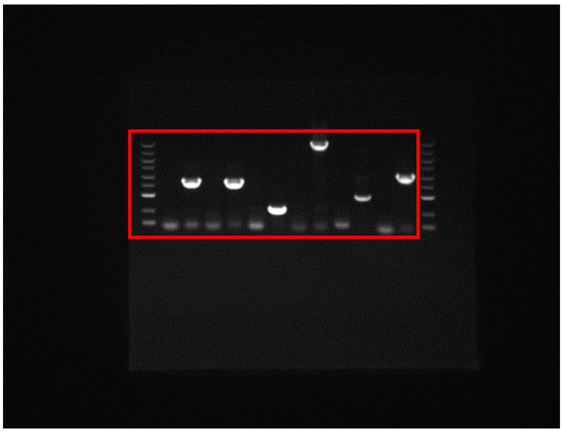

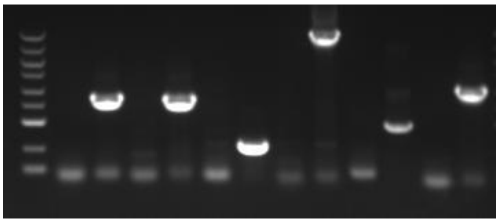

Supplementary Original data 3: Supplementary Figure 1

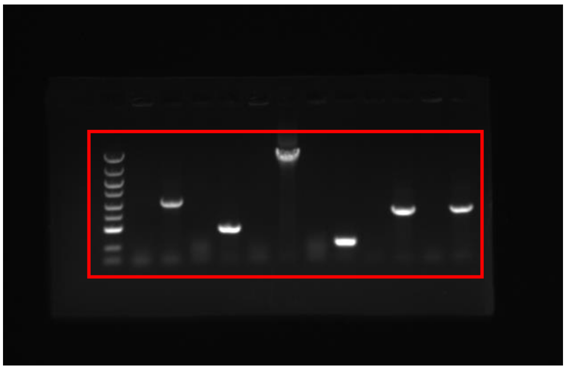

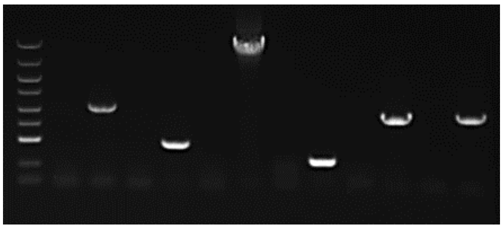

Supplementary Original data 4: Supplementary Figure 3A

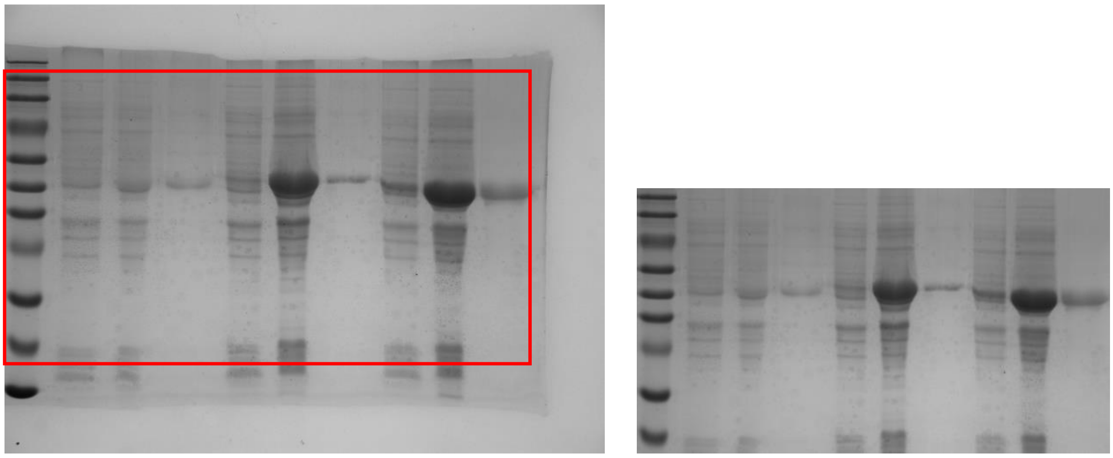

Supplementary Original data 5: Supplementary Figure 3B

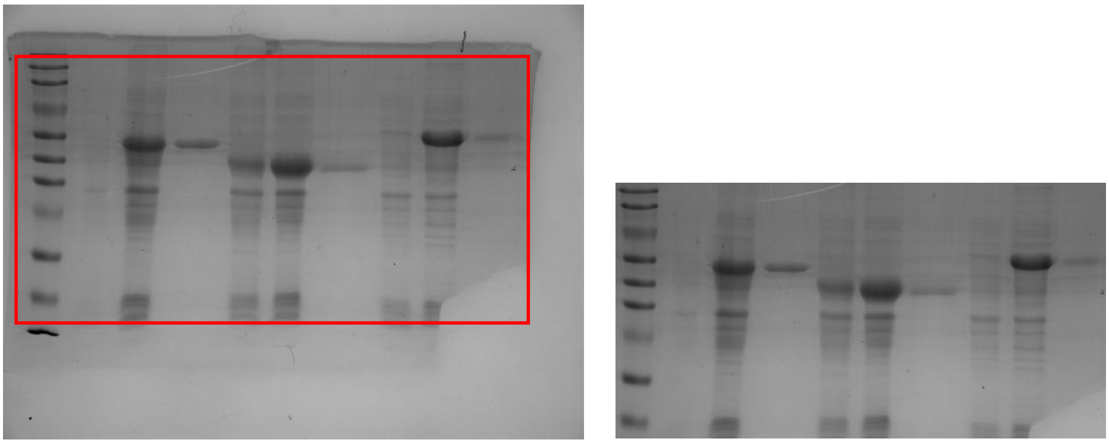

Supplementary Original data 6: Supplementary Figure 3C

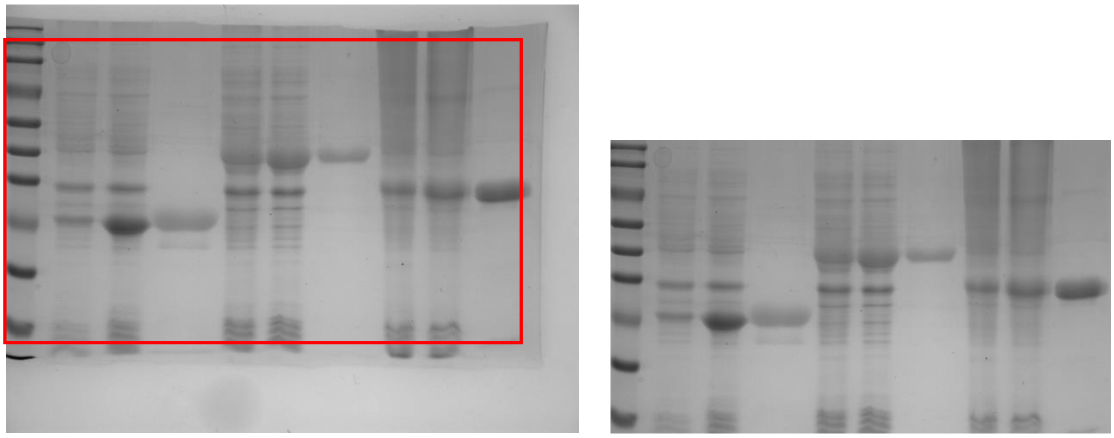

Supplementary Original data 7: Supplementary Figure 3D

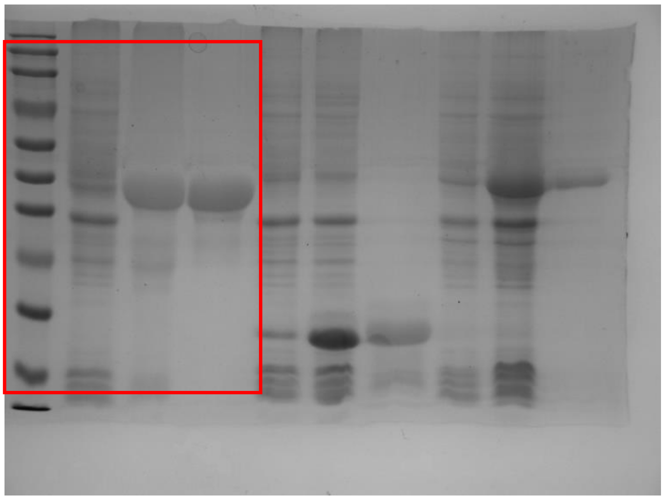

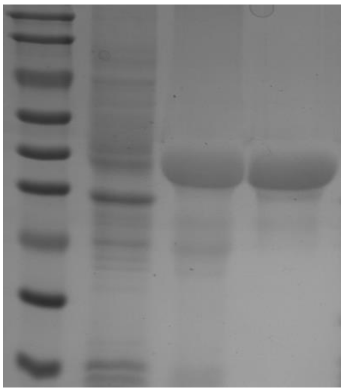

Supplementary Original data 8: Supplementary Figure 3E

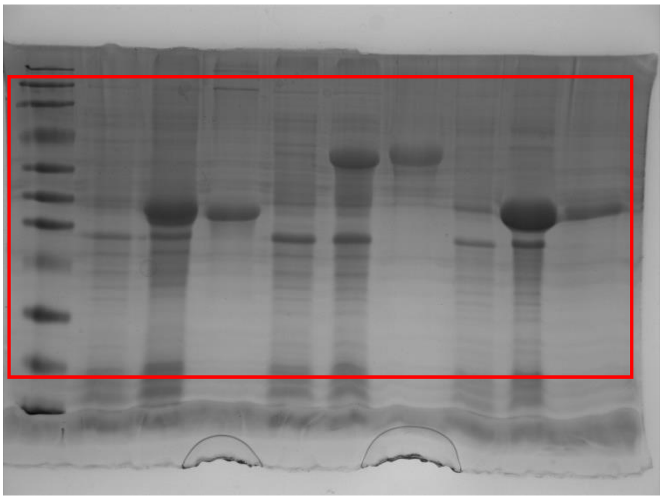

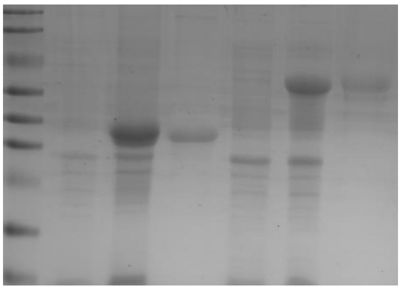

Supplement: Supplementary file 11 — Supplementary Material 11 [file 12917_2024_3948_MOESM11_ESM.pdf]
