# Supplementary material for: Attenuated vaccine PmCQ2Δ4555–4580 effectively protects mice against Pasteurella multocida infection
Source: BMC Vet Res. 2024 Mar 9;20:94. doi: 10.1186/s12917-024-03948-6 (PMC10924365; doi:10.1186/s12917-024-03948-6)
Supplement: Supplementary file 12 — Supplementary Material 12 [file 12917_2024_3948_MOESM12_ESM.pdf]

Attenuated vaccine PmCQ2△4555-4580 effectively protected mice against *Pasteurella multocida* infection

Fang He[a], Pan Xiong[a], Huihui Zhang[a], Liu Yang[a], Yangyang Qiu[a], Pan Li[b], Guangfu Zhao[a], Nengzhang Li[a]*, Yuanyi Peng[a]*

[a]College of Veterinary Medicine, Southwest University, Chongqing 400715, China

[b]Department of Environment and Safety Engineering, Taiyuan institute of technology, Taiyuan, 030008, China

***Corresponding author:** Nengzhang Li: lich2001020@163.com; Yuanyi Peng: pyy2002@sina.com.

Supplementary Figure legends

Supplementary Figure 1. The centrifugal state and stability of PmCQ2△4555-4580. A: The centrifugal state of PmCQ2, and PmCQ2Δ4555-4580 at 10000 rpm for 5 min. **B:** The stability detection of PmCQ2△4555-4580. M: 5000 DNA marker. Lanes 1, 3, 5, 7, 9, and 11: PmCQ2Δ4555-4580. Lanes 2, 4, 6, 8, 10, and 12: PmCQ2.

Supplementary Figure 2. The antibody titers of live PmCQ2△4555-4580. A: Scheme of immunization and antibody detection. **B:** The antibody titers represent the highest dilutions that produced positive results. The data of B were pooled from two independent experiments with 7 replicates per group.

Supplementary Figure 3. Immune protection of inactivated PmCQ2Δ4555-4580. A: Scheme of immunization and infection. **B and C**: The survival curve for immunized (inactivated PmCQ2 and PmCQ2Δ4555-4580) and control mice (n=8) following a challenge with PmCQ2 (3×10^7 CFU) **(B)** and PmB (3×10^7 CFU) **(C)**. The data were pooled from three independent experiments with 10 replicates per group.

Supplementary Figure 4. SDS-PAGE image of hypothetical immune protective proteins. A-D: The expressed and purified hypothetical immune protective proteins are verified by SDS-PAGE. a: non-induced bacteria culture (negative control); b: induced expressed hypothetical immune protective proteins; c: purified hypothetical immune protective proteins; M: Marker; 1: PmCQ2_004170; 2: PmCQ2_008190; 3: PmCQ2_008185; 4: PmCQ2_003710; 5： PmCQ2_000430; 6: PmCQ2_000440; 7: PmCQ2_002915; 8: PmCQ2_008205; 9: PmCQ2_010435; 10: PmCQ2_008725; 11: PmCQ2_000455; 12; PmCQ2_004485.

Supplementary Figure 5. The immune protection of other hypothetical immune protective proteins. A and C: The antibody titer of immunized (immunoprotective antigens) and control mice (n=8) following a challenge with PmCQ2 (3×10^7 CFU). **B**

and D: The survival curve for immunized (immuno-protective antigens) and control mice (n=8) following a challenge with PmCQ2 (3×10^7 CFU). The data of C and D were pooled from two independent experiments with 10 replicates per group.

Supplementary Figure 6. The expression of virulence genes in PmCQ2Δ4555-4580. **A-B:** Capsular polysaccharide related DEGs in RNA-seq **(A)** and in RT-qPCR (n=3) **(B)**. **C-D:** LPS synthesis related DEGs in RNA-seq **(C)** and in RT-qPCR (n=3) **(D)**. **E-F**: OMPs related DEGs in RNA-seq **(E)** and in RT-qPCR (n=3) **(F)**. B, D, and F were representative of two independent experiments with 3 replicates per group and analyzed by multiple comparative analysis, and expressed as means ±SD (*$p < 0.05$, ** $p < 0.01$, *** $p < 0.001$).
